# The molecular basis for sarcomere organization in vertebrate skeletal muscle

**DOI:** 10.1016/j.cell.2021.02.047

**Published:** 2021-04-15

**Authors:** Zhexin Wang, Michael Grange, Thorsten Wagner, Ay Lin Kho, Mathias Gautel, Stefan Raunser

**Affiliations:** 1 Department of Structural Biochemistry, Max Planck Institute of Molecular Physiology, Otto-Hahn-Strasse 11, 44227 Dortmund, Germany; 2 The Randall Centre for Cell and Molecular Biophysics, School of Basic and Medical Biosciences, Kings College London BHF Excellence Centre, New Hunt’s House, Guy’s Campus, London SE1 1UL, UK

**Keywords:** muscle, sarcomere, actin, myosin, Z-disc, tropomyosin, electron tomography, structure

## Abstract

Sarcomeres are force-generating and load-bearing devices of muscles. A precise molecular picture of how sarcomeres are built underpins understanding their role in health and disease. Here, we determine the molecular architecture of native vertebrate skeletal sarcomeres by electron cryo-tomography. Our reconstruction reveals molecular details of the three-dimensional organization and interaction of actin and myosin in the A-band, I-band, and Z-disc and demonstrates that α-actinin cross-links antiparallel actin filaments by forming doublets with 6-nm spacing. Structures of myosin, tropomyosin, and actin at ~10 Å further reveal two conformations of the “double-head” myosin, where the flexible orientation of the lever arm and light chains enable myosin not only to interact with the same actin filament, but also to split between two actin filaments. Our results provide unexpected insights into the fundamental organization of vertebrate skeletal muscle and serve as a strong foundation for future investigations of muscle diseases.

## Highlights

• Three-dimensional sarcomere organization and plasticity at the molecular level
• Myosin double heads can adopt two different interactions with actin filaments
• Transition between tropomyosin states happens within one tropomyosin unit
• An irregular mesh of α-actinin doublets cross-links antiparallel actin filaments

## Graphical abstract

**Figure:**
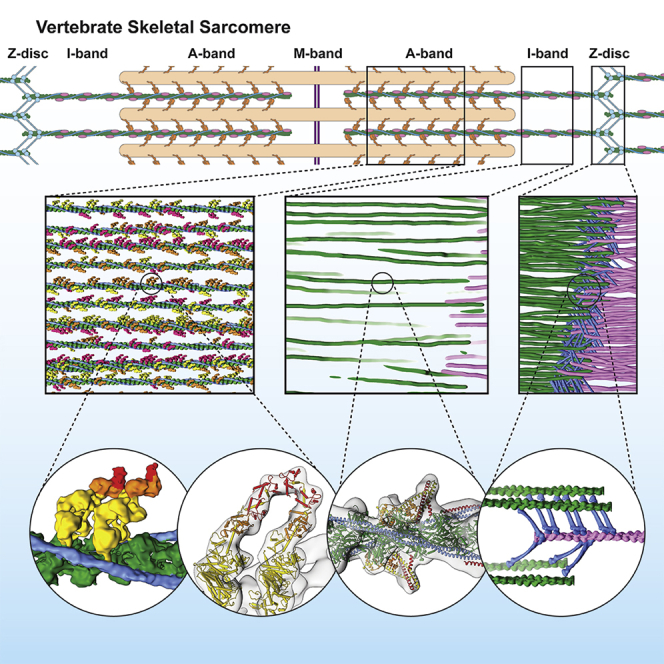

## Teaser

Visualizing the mouse sarcomere in the rigor state using electron cryo-tomography reveals architectural details of the different zones and provides insight into how key factors are arranged within them to support function during muscle contraction.

## Introduction

Skeletal muscle is an essential tissue required for efficient movement in vertebrates. In humans, dysfunctional muscle function leads to a broad variety of physiological disorders, ranging from common muscle cramps to severe myopathies (Bönnemann and Laing, 2004; Savarese et al., 2020). In muscle, specialized cells align and assemble during development into a fundamental unit known as the muscle fiber. Individual muscle fibers can be very large syncytia of up to several centimeters in length and are comprised of the force-generating and load-bearing devices of muscles called sarcomeres.

Each sarcomere can be axially divided into different zones based on its ultrastructure: the I (isotropic)-band where only actin (thin) filaments are present, the A (anisotropic)-band that contains overlapping actin and myosin (thick) filaments, the Z-disc that borders the sarcomere at its ends, anchoring the barbed (+) ends of actin filaments, and the M-band in the center, where only myosin filaments are present and cross-linked. The Z-disc is formed by the ends of antiparallel thin filaments from adjacent sarcomeres. These filaments are laterally cross-linked by α-actinin (Ribeiro et al., 2014; Takahashi and Hattori, 1989), forming an intricate network with other proteins such as titin, nebulin, and myotilin (Gontier et al., 2005; Gregorio et al., 1998; Lange et al., 2006). In the M-band, myosin filaments are anchored in a hexagonal array by a complex of the proteins myomesin, titin, and obscurin/obsl1. The network formed by these proteins and myosin filaments provide mechanical stability for the sarcomere by absorbing misbalanced longitudinal force produced by myosin heads. Deformation of the M-band by shear forces could allow it to act as a strain sensor and thus play an important role in signaling (Gautel, 2011; Gautel and Djinović-Carugo, 2016; Lange et al., 2020).

The myosin filaments are stably linked to actin filaments by a third “connecting” filament, consisting of the gigantic titin protein. Titin’s N terminus is bound to α-actinin, telethonin, and possibly CapZ at the Z-disc (Gautel and Djinović-Carugo, 2016; Ribeiro et al., 2014). Its C terminus interacts with myomesin and obscurin at the M-band (Gautel, 2011; Gautel and Djinović-Carugo, 2016).

The basis of the mechanism of skeletal muscle action is the cyclical interaction of actin and myosin, converting chemical into kinetic energy, which leads to relative movement between thin and thick filaments and is the primary driver of force generation within the sarcomere (Huxley and Hanson, 1954; Huxley and Niedergerke, 1954).

Images of fixed, stained, and sectioned sarcomeres acquired using electron microscopy revealed a hexagonal arrangement of thick and thin filaments and cross-bridges between them formed by myosin head domains in the A-band (Hirose and Wakabayashi, 1993; Huxley, 1957; Tsukita and Yano, 1985). The composition, regulation, and function of arthropod and vertebrate muscles differ in key aspects (Bullard and Pastore, 2011; Taylor et al., 2007). In vertebrate skeletal muscle, one thin filament interacts with three neighboring thick filaments instead of two, leading to a more complicated model of cross-bridge formation (Hooper et al., 2008; Luther and Squire, 2014). In insect flight muscle sarcomeres, the three-dimensional structure of the cross-bridges were further analyzed using electron tomography of plastic-embedded samples (Schmitz et al., 1996, 1997; Taylor et al., 1984) and freeze-substituted samples (Liu et al., 2004; Wu et al., 2010), showing the distribution and overall shapes of cross-bridges in different states. However, these studies were limited in resolution and did not allow an understanding of the molecular detail of cross-bridges in the A-band. Similarly, a three-dimensional tomographic study of the vertebrate sarcomere showing the variable lengths of thin filaments in different vertebrate muscle types (Burgoyne et al., 2008) was also limited due to the applied method. Therefore, the molecular details underpinning the organization of the vertebrate skeletal muscle sarcomere remain elusive.

Z-discs are complex structures despite their fundamentally “simple” function as stable yet flexible crosslinkers of antiparallel actin filaments (Ribeiro et al., 2014). The core unit is the actin-α-actinin link, crosslinking actin filaments at roughly 90° turns in variable layers of between 2 to 7 (Luther, 2009). Images of the transverse section of Z-discs of chemically fixed mouse soleus and cardiac muscle showed two types of appearance in response to, and absence of, stimulation: a relaxed “small square” form and an active “basket-weave” form (Goldstein et al., 1990). Both states exhibit a square arrangement of thin filaments. Due to the lack of a 3D visualization of the I-band thin filaments, it remains obscure as to how the thin filaments progress from a hexagonal pattern in the A-band to a square pattern in the Z-disc.

The actin-α-actinin crosslinks are known to be conformationally flexible and can be affected by the activation state of the thick filament as well as temperature and ionic strength (Perz-Edwards and Reedy, 2011). The conformational plasticity of the Z-disc is believed not only to provide resistance to mechanical forces during contraction and relaxation but also to translate mechanical stress into biochemical signals via a number of signaling pathways (Pyle and Solaro, 2004) involving transiently associated Z-disc proteins.

The recently presented structure from resin-embedded sections of the highly atypical Z-disc of the midshipman sonic muscle, structures that are at least 10 times thicker than the Z-discs of representative vertebrate skeletal muscles, revealed a fundamental arrangement of transverse crosslinks with an axial displacement of 19.2 nm, in line with previous work at lower resolution (Burgoyne et al., 2015, 2019; Perz-Edwards and Reedy, 2011). A further study of the Z-disc from isolated branches of cardiac myofibrils using electron cryo-tomography (cryo-ET) determined averaged structures of actin-α-actinin complexes in two states (putatively, the relaxed and activated states) at ~23 Å resolution (Oda and Yanagisawa, 2020). These two structures highlight changes within the Z-disc in response to torque applied by the myosin heads on thin filaments, whereby α-actinin can pivot via linker domains between the central “rod” domain and the N-terminal actin binding domain. Nevertheless, any inherent strain within a given sarcomere was not ascertained within these data and, as the structures are averaged from many individual sub-volumes, any heterogeneity of α-actinin arrangement within the same Z-disc or across different Z-discs remains undiscovered. The current molecular understanding of actin-myosin interactions and the function and mechanism of other essential sarcomeric proteins, such as troponin, titin, and α-actinin has been derived from atomic structures of *in vitro* reconstituted components by X-ray crystallography, single particle cryo-EM (Behrmann et al., 2012; von Castelmur et al., 2008; von der Ecken et al., 2016; Mentes et al., 2018; Ribeiro et al., 2014; Yamada et al., 2020) or ensemble information, obtained from X-ray diffraction of muscle fibers (Al-Khayat and Squire, 2006; Al-Khayat et al., 2003; Huxley and Brown, 1967; Lombardi et al., 2004; Squire et al., 2004). Although these structures serve as a foundation for determining interactions among different sarcomeric components, high-resolution information on their biological arrangement in the context of a sarcomere is lacking. As such, an *in situ* approach that allows a molecular and structural analysis of a functional sarcomere is necessary for understanding the native interactions and localization of sarcomeric proteins.

The major obstacles in obtaining molecular structural details of the sarcomere using conventional tomographic methods lie in the artifacts arising from fixation and staining methods, dehydration of the sample, the diamond blade during sectioning, or from the shrinkage and deformation of plastic sections during imaging (Bennett, 1974). All these factors impede the determination of high-resolution features of macromolecules within the sample. In the past 15 years, investigations of the structure of muscle fibers with conventional electron tomography have provided important insights into the sarcomeric actomyosin cross-bridge architecture (Liu et al., 2004; Wu et al., 2010). However, chemical fixation, freeze-substitution and EM hardware limited the obtainable resolution.

To circumvent these artifacts, cryo-focused ion beam-milling (cryo-FIB) (Marko et al., 2007; Rigort et al., 2012; Schaffer et al., 2017) of vitrified samples has been introduced, facilitating direct imaging via cryo-ET of cellular sections with minimal artifacts and in a frozen-hydrated state (e.g., Hagen et al., 2015; Mahamid et al., 2016; Weiss et al., 2019). As these techniques preserve the hydrogen bond network and fine ultrastructure of samples, they allow the subsequent structure determination using sub-volume averaging after cryo-ET. Recent developments in the field have led to a number of structures at resolutions approaching the atomic scale derived from *in situ* environments and have demonstrated the ability to inform on the molecular and structural details of proteins in their native state and context (Schur et al., 2016; Tegunov et al., 2020). Cryo-FIB and cryo-ET were recently employed to investigate neonatal rat cardiomyocytes and provided an initial insight into the organization of muscular filaments inside immature sarcomeres, in which the thick filaments and thin filaments could be observed in a semi-striated arrangement (Burbaum et al., 2020). However, the details of a mature sarcomere from a fully developed vertebrate myofibril, wherein different zones and structures can be distinguished, remain elusive.

Combining cryo-FIB, cryo-ET, and sub-volume averaging, we present the three-dimensional structure of a mouse psoas sarcomere in the rigor state with a comprehensive description of the organization and interaction of key sarcomeric proteins in molecular detail.

## Results and discussion

### Vitrified myofibrils exhibit undisturbed sarcomere ultrastructure in molecular detail

**Figure S1:**
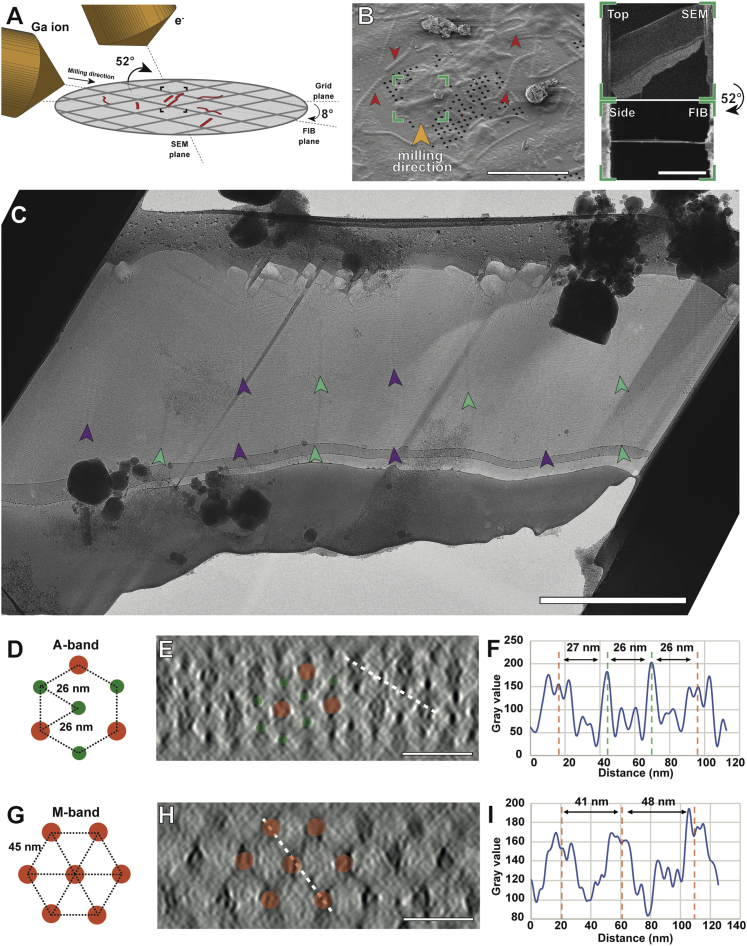
Cryo-FIB-ET of isolated mouse myofibrils reveals sarcomere ultrastructure and hexagonal arrangement of filaments in the cross-section view, related to Figure 1 (A) Cartoon showing the arrangement of the SEM and FIB ion source with respect to the grid. Myofibrils are depicted in red. (B) Left: SEM image taken 45° to the plane of a grid on which myofibrils were vitrified, with red arrow heads pointing toward the myofibrils. The orange arrow head indicates the milling direction. Scale bar, 50 μm. Right: Top (taken with electron beam) and side (taken with ion beam) views of the lamella taken after milling in the FIB-SEM. The top view was taken 60° angle to the plane of the grid. The side view was taken 8° to the plane of the grid, which was also the milling angle. Scale bar, 5 μm. (C) Projection image of a lamella after FIB-milling recorded at a TEM at stage 0°. Purple arrow heads indicate M-bands of sarcomeres, while green arrow heads indicate Z-discs. Scale bar, 3 μm. (D and G) Schematic diagrams of the triangular arrangement of thick filaments in the A-band (D) and the hexagonal arrangement of thin filaments in the M-band (G). Thin and thick filaments are represented by green and orange circles, respectively. (E and H) Slices of the cross-section view of tomograms in the A-band (E) and the M-band (H). The tomograms were previously filtered with an equator filter on XY slices in Fourier space to remove signals from cross-bridges. The thicknesses of lamellae were measured in this view. Scale bar, 50 nm. (F and I) Distances between filaments were measured using the line profiles of the white dotted lines in (E) and (H), respectively.

In order to investigate the native organization of an intact sarcomere, we vitrified mouse psoas myofibrils in the rigor state (i.e., without ATP) by plunge-freezing in liquid ethane and subsequently prepared thin lamellae using cryo-FIB (Tacke et al., 2020; STAR Methods). The lamellae were 30–150 nm thin and ideally suited for cryo-ET (Figure S1).

**Figure 1:**
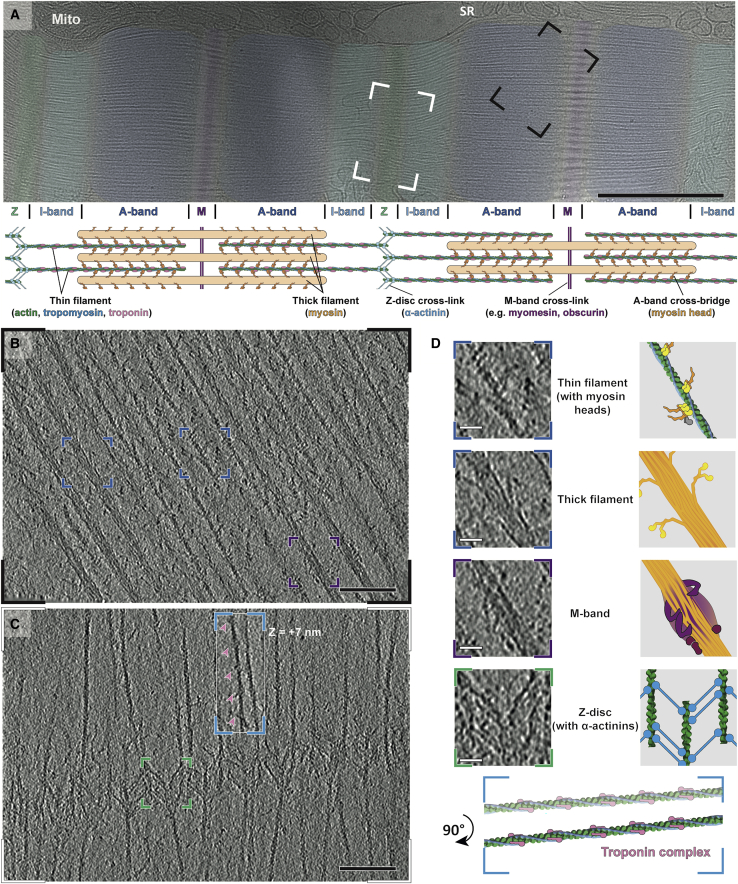
Isolated mouse skeletal myofibrils imaged using electron cryotomography (A) Projection image of mouse skeletal muscle, with Z-disc, I-, M-, and A-bands visible (green, light blue, dark blue, and purple, respectively). A schematic diagram is shown below, highlighting the lateral organization and cross-links in different zones. Mitochondria (Mito) and sarcoplasmic reticulum (SR) can be identified between sarcomeres. Scale bar, 1 μm. (B) Slice through an electron cryo-tomogram spanning A- to M-band. Its representative position on a sarcomere is marked as the black box in (A) (not same sarcomere). In this region, myosin heads bound to the thin filament, myosin tails emanating from the thick filament (dark blue insets) and obscure protein densities at the M-band (purple inset) can be discerned. Scale bar, 100 nm. (C) Slice through an electron cryo-tomogram spanning I-band and Z-disc. Its representative position on a sarcomere is marked as the white box in (A) (not same sarcomere). The tail-feather-like arrangement of α-actinin molecules cross-linking thin filaments in a zig-zag manner is visible (green inset). Thin filaments in the I-band have regularly-spaced nodes corresponding to troponin complexes (pink arrow heads, blue inset). A slice of the same location but 7 nm above from the rest of the slice is shown inset. Scale bar, 100 nm. (D) Larger view of insets described in (A)–(C), with cartoon depictions of densities. Scale bar, 20 nm. See also Figure S1.

Vitrified myofibrils show an intact sarcomere ultrastructure, including apparent Z-discs, I-bands, A-bands, M-bands, and organelles such as mitochondria and sarcoplasmic reticulum localized between laterally adjacent sarcomeres (Figures 1A and S1). Importantly, the tomograms include structures that could not previously be visualized *in situ*. Of particular note are myosin double heads forming regular cross-bridges that resemble an “arrow-head,” thin myosin tails mainly composed of coiled-coil structures (Chew and Squire, 1995) protruding from the thick filament, and details of the Z-disc and troponin complexes (Figures 1B–1D). The M-band consists of numerous structural proteins other than myosin, such as myomesin, titin, and obscurin as well as accessory proteins with transient localization, such as FHL-2, Nbr1, MURFs, or calpain-3 (Lange et al., 2006, 2020). Together, they form pleomorphic densities decorating and cross-linking the thick filaments (Figures 1B and 1D). This heterogeneity hampers identification of individual proteins in this crowded region. Therefore, we focused on the details of the A-band, I-band, and Z-disc in the following sections.

It is well-known from cross-sectional views of myofibrils that thin and thick filaments are hexagonally arranged in the A-band (Huxley, 1957). Interestingly, in immature sarcomeres found within neonatal cardiomyocytes, the hexameric pattern is limited to thick filaments, suggesting that thin filament organization is a later process of sarcomeric maturation *in vivo* (Burbaum et al., 2020). In orthogonal views of our tomograms, we can clearly discern this hexagonal pattern in the A-band, demonstrating that our preparations are consistent with previous studies (Figures S1D–S1F). One thin filament is surrounded by three thick filaments and three other thin filaments at a distance of around 26 nm (Figures S1D–S1F). Thick filaments form triangular patterns with an average inter-thick-filament distance of 45 nm (Figures S1G–S1I). This arrangement of thick filaments is maintained from the A-band to the M-band where thin filaments are missing.

### *In situ* actomyosin structure reveals a myosin double-head with different lever arm conformations

**Figure S2:**
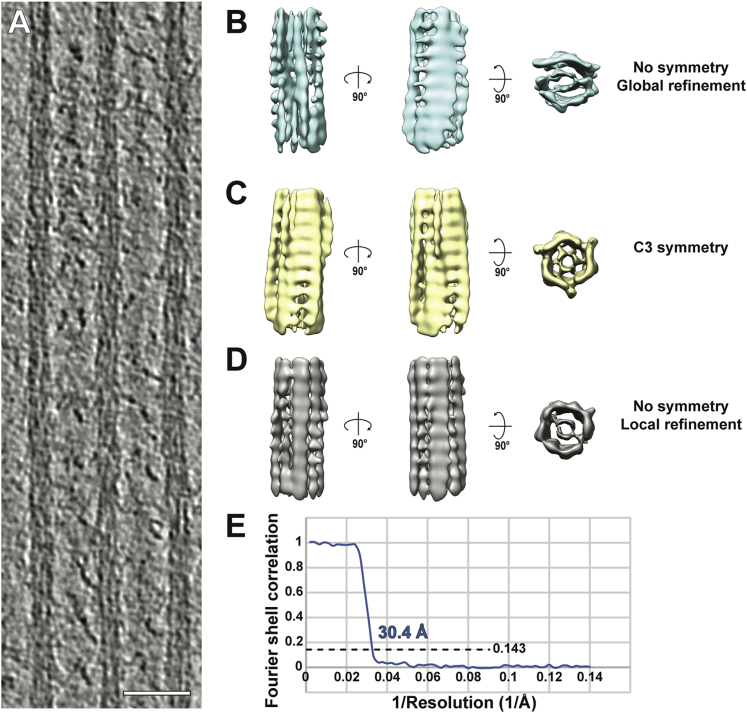
Sub-volume averaging of thick filaments, related to Figures 3 and 5 (A) Slice through a tomogram depicting three adjacent thick filaments. Scale bar, 50 nm. (B) Averaged structure from a global refinement. The map suffers from missing wedge artifacts, indicating alignment focused on the missing wedge instead of actual structural features. (C) The distribution of myosin heads when retracted to the thick filaments in the relaxed state has a C3 symmetry. We therefore performed a refinement and reconstruction applying C3 symmetry. However, this did not improve the quality of the map, indicating that the core of vertebrate thick filaments is not C3 symmetric. (D) A local refinement with restricted possible rotation angles reduced alignment errors and thereby missing wedge artifacts. This reconstruction was used as a model for thick filament in Figures 3 and 5. (E) The estimated resolution of the reconstruction in (D) is 30.4 Å using the 0.143 criterion.

Segmentation of the tomograms enabled us to unequivocally characterize the 3D arrangement of thin and thick filaments and the cross-bridges within a sarcomere (Video S1). To increase the level of observable detail that we could resolve with our data, we employed sub-volume averaging to determine the structure of the thin and thick filament and cross-bridges. The reconstruction of the thick filament was proved to be difficult due to its heterogeneity, flexibility, and an absence of obvious symmetry. Nevertheless, we obtained a low-resolution reconstruction showing the architecture of the core of thick filaments that we could use for the subsequent analyses (Figure S2).

Video S1. From a tomogram to the molecular model of the A-band, related to Figures 3 and 5 and STAR Methods Slices of a tomogram depicting the A-band of a sarcomere are shown by moving through the Z axis. Thick (red and yellow colors) and thin filaments (green and blue colors) were annotated in 3D. The actin-tropomyosin structure fully decorated with myosin heads was then rigid-body-fitted in the annotated volume of each thin filament (gray). Myosin heads mostly outside the density were removed and only the heads that are bound to filaments are shown in the final model and colored based on the thick filament they originate from.

**Figure S3:**
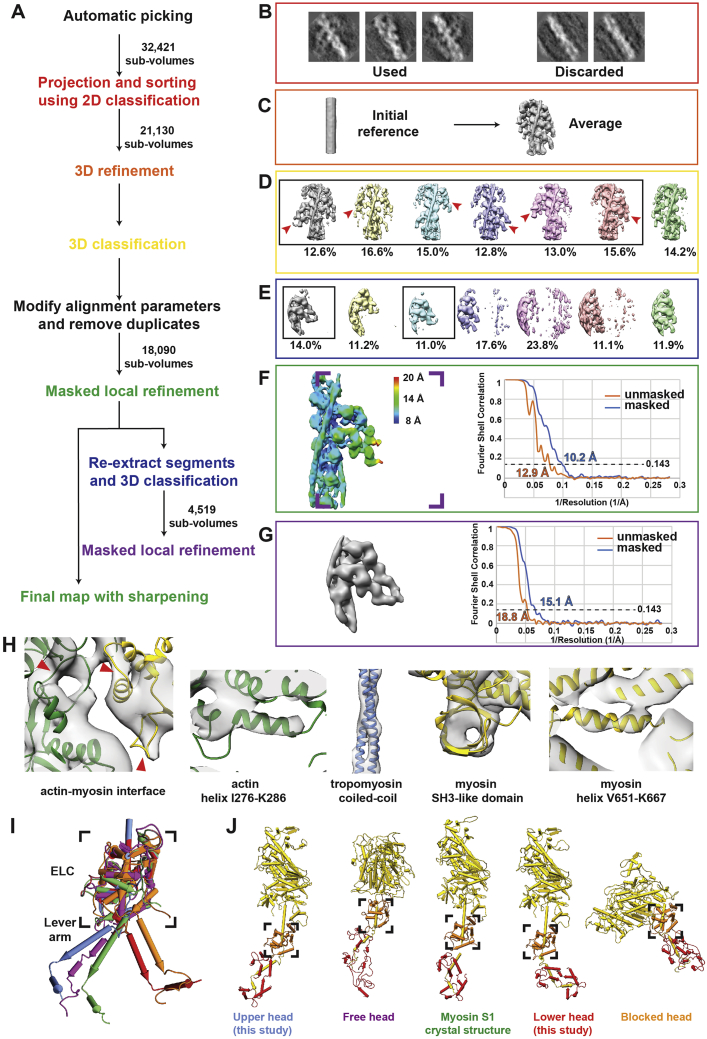
Strategies of sub-volume averaging of the *in situ* actomyosin complex and different “kink” conformations in the lever arm, related to Figure 2 and STAR Methods (A) Workflow of sub-volume averaging leading to the final structures of the *in situ* actomyosin complex and double-head myosin. Details of the colored steps are shown in (B-G). (B) Examples of selected and discarded 2D class averages. After cleaning by 2D sorting, 21,130 out of 32,421 sub-volumes were selected for subsequent processing. (C) Initial reference and the average after the first 3D refinement with only a spherical mask with a diameter of 340 Å applied. (D) Classes of the actomyosin complex after 3D classification. Prominent myosin double head pairs in each class are indicated by red arrow heads. These pairs were later aligned to each other during merging the classes by modifying the alignment parameters. The classes in the black box were selected and used for subsequent processing. (E) Classes of the myosin double-heads after the 3D classification applied on the sub-volumes re-centered on myosin heads. The two classes in the black boxes showing clear densities for the double-head were selected for the final averaging. (F) Left: final reconstruction of the actomyosin complex generated from the local refinement with a mask around the thin filament and a pair of myosin heads after merging the classes in (D). The map is colored based on local resolution. The purple inset shows the area of the re-centered sub-volumes for double-head analysis in (E) and (G). Right: The estimated resolution of the reconstruction is 10.2 Å using the 0.143 criterion. (G) Left: final average of the complete myosin double-head including RLC. Right: The estimated resolution of the reconstruction is 15.1 Å using the 0.143 criterion. (H) Example details showing secondary structures that are visible in the averaged structure in F. The red arrow heads indicate the D-loop of actin, helix-loop-helix motif and loop 3 of myosin from left to right at the interface between actin and myosin. (I) Alignment of ELCs, together with the ELC-binding region, from different atomic models of myosin heads. Only the RLC-binding helix of the lever arm in each model is shown instead of the complete RLC for clear visualization. Two different groups of conformations are shown. The conformation of the lower head (red) is similar to the relaxed blocked state (orange). The upper head (blue) exhibits a similar conformation to the relaxed free head (purple) and the crystal structure of squid myosin S1 (green). (J) Side-by-side comparison of the complete myosin atomic models in (I) showing the heavy chain and both light chains.

**Figure 2:**
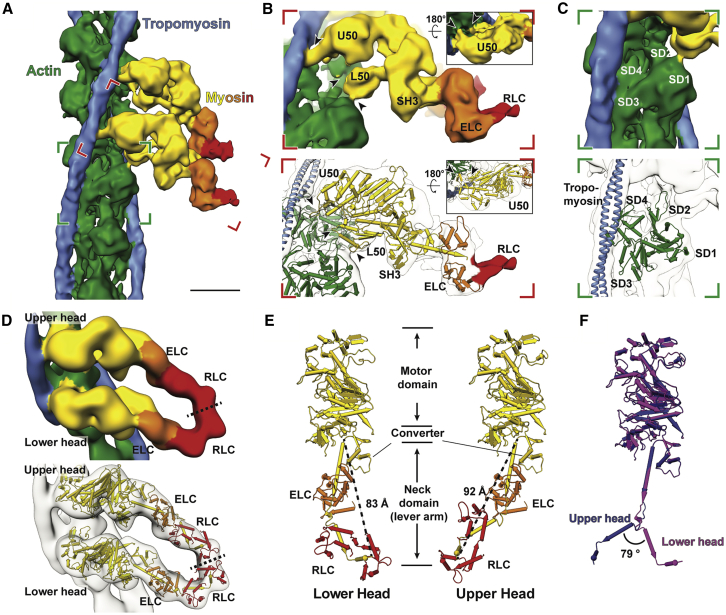
Sub-volume averaging of thin filaments reveals the interaction of a double-head myosin with the thin filament and two conformations of light chain domains within a double-head (A) Surface view of the *in situ* actomyosin structure, showing actin filament (green), tropomyosin (blue), and myosin double heads, with motor domains (yellow), essential light chains (orange), and regulatory light chains (red). Scale bar, 5 nm. (B) Close-up view of the lower myosin head and homology models based on PDB: 3I5G, 5JLH, and 6KN8. The upper 50 kDa (U50), lower 50 kDa (L50), SH3, and essential light chains (ELC) can be allocated in the map, along with part of the regulatory domain (RLC). Arrow heads indicate the interaction interfaces between actin and myosin at loop 4, helix-loop-helix motif, loop 3 of myosin (top to bottom). Arrow heads in the inset depict interaction interfaces at the cardiomyopathy loop and loop 2 (left to right). (C) Close-up of an actin subunit and structural model fitted into the EM map showing the four subdomains of an actin subunit (SD1–SD4). (D) Surface view of the structure of a complete myosin double-head including RLCs determined from averaging shifted sub-volumes (see Figure S4). Their interface is indicated by a dotted line. (E) Comparison between the lower and upper heads within one double head, showing two different conformations in the lever arm that interacts with RLC and ELC. Lengths of the lever arms were measured between G772 and L844. (F) Alignment of the lower (purple) and upper (blue) heads heavy chain, showing two different kinks between the ELC-binding region and the RLC-binding region. See also Figures S2 and S3.

The three-dimensional reconstruction of the thin filament and cross-bridges could be resolved to a higher resolution. Segmentation of the tomograms and 3D classification of all sub-volumes of the cross-bridges indicated that the two heads from the same myosin molecule, known as double-heads, mostly bind to two neighboring actin subunits (Figure S3D). Hence, we merged multiple classes after modifying alignment parameters to obtain a structure of a native thin filament with the myosin double-head bound (Figure 2A; STAR Methods). The reconstruction has a resolution of 10.2 Å and we could clearly assign tropomyosin and the domains of actin and of the myosin heads and necks, including the essential chains (ELC) and part of the regulatory light chains (RLC) (Figures 2A and S3; STAR Methods).

We calculated a homology model using the rigor cytoplasmic actomyosin cryo-EM structure (PDB: 5JLH) (von der Ecken et al., 2016) and the crystal structure of the S1 fragment from squid muscle (PDB: 3I5G) (Yang et al., 2007) as a template and fitted it into the density map using rigid body fitting (Figures 2B and 2C). We chose this myosin structure because there are no crystal structures of a complete vertebrate myosin S1 fragment in the rigor state available. The model fits well into the density, demonstrating that the *in vitro* structures resemble well the structures determined *in situ*. At this resolution, both myosin heads bind to the actin filament in a structurally identical manner with close interactions formed between actin subunits and myosin as well as tropomyosin (Figures 2B and 2C). Both ELCs bind to the upper parts of the lever arms (residues L788–L800) as is seen in the crystal structure. The density threshold corresponding to the RLCs and RLC binding domains after averaging is considerably lower than the rest of the structure, indicating a higher flexibility or variability in this region.

To improve the quality of the reconstruction in the region of the RLC, we shifted the center of sub-volume averaging from the thin filament to the myosin head. Through further classification, we determined a structure of the complete double-head myosin S1 fragment, including the two heavy chains that are bound to actin, two ELCs and two RLCs (Figure 2D). The S2 fragment that tethers myosin heads to the thick filament was not resolved in this structure, likely due to the different positions and angles it takes in the sarcomere.

Although a homology model of the myosin motor domain and ELC of the squid myosin crystal structure (PDB: 3I5G) fits well into our density, the RLCs and the lower part of the lever arm (residues R811–L844) had to be fitted separately (Figure 2D). Compared with its conformation in the crystal structure, the lever arm is strongly kinked between the ELC and RLC binding regions (residues M801–E810), resulting in a bent conformation (Figure 2E). Interestingly, the kink in the upper head is in the opposite direction compared to the one in the lower head, bringing the RLCs of the two heads in close proximity (Figure 2F). This crucial observation reveals the ability of the myosin double head to accommodate force production, and has not been validated structurally in any previous study in any system. It is also consistent with previous cross-linking and FRET experiments, which suggested a small distance between the two RLCs in a double head (Bower et al., 1992; Chakrabarty et al., 2002; Chantler et al., 1991).

This arrangement, which is probably stabilized by interactions between the ELCs and RLCs, allows for the simultaneous binding of the two heads to actin and at the same time brings their necks close enough together to continue with the coiled-coil structure of the joint S2 fragment. In addition, the opposite direction of the kinks on the two lever arms leads to a similar length of the two arms. This is important because the length of the lever arm determines the step size of myosin heads on actin (Ruff et al., 2001).

In this context, it is interesting that the hinge in the lever arm together with a hook (residues W824–W826 in molluscan myosin) have previously been shown to be important for regulated myosins (Brown et al., 2011; Himmel et al., 2009). In this case, the light chains control myosin activity even in the absence of actin (Wells and Bagshaw, 1985) and the presence of Ca^2+^ determines the stability of the ELC/RLC interface. Different ELC/RLC interfaces were also suggested from the latest model of myosin in the relaxed state, where the heads fold back to the thick filament (Hu et al., 2016; Knupp et al., 2019). Notably, the arrangement of the ELC/RLC of the lower head in our model resembles that in the “blocked” head of the off-state myosin while the upper head ELC/RLC is similar to the “free” head (Figures S3I and S3J). This suggests that there are only two possible angles for the kink in the lever arm, which are likely to be determined by the interaction of the ELCs and RLCs. This interaction probably stabilizes the two conformations to rigidify the lever arm, which is needed for the proper transmission of the force of the power stroke (Holmes, 1997). It will be interesting to visualize how the phosphorylation state of the RLC can modulate the interaction of the ELCs and RLCs and thus modulate muscle contractility.

Various structures for the single isolated myosin head have been determined in different states of the ATP hydrolysis cycle (e.g., Behrmann et al., 2012; von der Ecken et al., 2016; Houdusse et al., 2000; Mentes et al., 2018; Risal et al., 2004; Yang et al., 2007). Furthermore, whereas the myosin double-head has also been determined in structures of isolated thick filaments and in 2D crystals of heavy meromyosin (Alamo et al., 2008; Baumann et al., 2012; Knupp et al., 2019; Wendt et al., 2001), our *in situ* actomyosin complex is a structure of the myosin double-head interacting with the thin filament determined directly within fully organized myofibrils. Our analysis of this structure can be used to further inform other states of the myosin double-head, such as the pre-power stroke state and the relaxed state, to further understand the cross-bridge cycle (Spudich, 2001) within a sarcomere in a native context.

### Cross-bridges in the A-band depict a pseudo-regular distribution of myosin heads and reveal a split-head conformation

**Figure 3:**
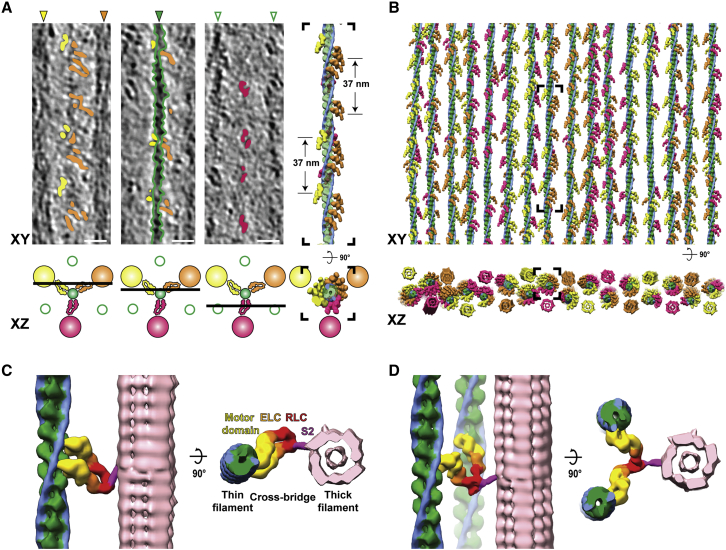
Organization of the A-band in natively isolated myofibrils shows that myosin heads can adopt two interactions with thin filaments (A) XY-slices through a tomogram at three different Z positions (illustrated by the cartoon below), highlighting the myosin head densities (orange, yellow, and magenta) and the thin filament (green) apparent within the volume. Annotation of densities produces a volume in which filtered actomyosin can be fitted, shown on the right and in (B). The three myosin colors represent the contribution of the myosin head from a respective neighboring thick filament. Scale bars, 20 nm. (B) Fitted model of all thin filaments and myosin heads in a tomogram, with the black inset depicting the filament shown in (A) In the XZ view, reconstructions of corresponding thick filaments are also shown. (C) A typical cross-bridge with a myosin double-head. (D) A rare myosin split-head with two heads from the same myosin molecule binding to two different actin filaments. See also Figures S2 and S4 and Video S1.

**Figure S4:**
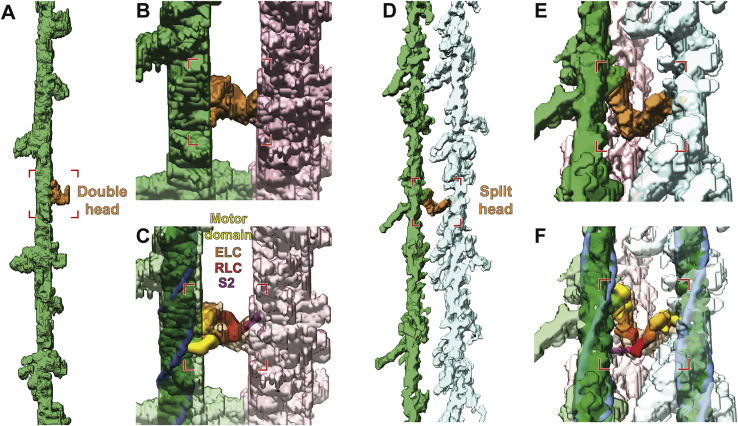
Annotation and fitting of split and double heads of myosin between thin and thick filaments from A-band tomograms, related to Figures 3 and 5 (A) Annotated density of a thin filament including the myosin heads (green), with the annotated density for a myosin double-head highlighted in orange. (B) Oblique and zoomed-in view of the double-head density and its associated thick filament segmentation (pink). (C) Same view as in (B) including the fitted thin filament and myosin double-head models from sub-volume averaging. (D) Annotated density of two thin filaments including the myosin heads (green and light blue), highlighting annotated density corresponding split-head in orange. (E) Oblique and zoomed-in view of the split-head density and its associated thick filament segmentation. (F) Same view as in (E) including the fitted thin filament and myosin split-head models. The split-head is composed of two heads in the upper head conformation in order to be fitted into the density.

In order to identify the proportion of myosin heads that bind to thin filaments in a rigor state sarcomere, we annotated and isolated the densities corresponding to the cross-bridges as well as thick and thin filaments in the A-band (Figures 3A and S4). Combining annotated filaments with structures determined via sub-volume averaging allowed us to produce a molecular map of the thin and thick filaments and attached myosin heads (Figures 3B and S4).

Of the 30 thin filaments annotated containing 5,664 actin subunits, 2,734 myosin heads were fitted into the cross-bridge densities, corresponding to 82.5% of the total number of myosin heads in this region calculated from the theoretical number of myosin heads per given length of the thick filament (6 heads per 14.3 nm, 294 heads per thick filament [Bennett et al., 2020], total heads in our analysis = 3,315). This suggests that not all myosin heads are attached to thin filaments, even in the rigor state.

Similar to sarcomeres from insect muscle, all thin filaments are in helical register (Figure 3B) and cross-bridges appear at regular target regions every ~37 nm on a thin filament between each pair of thick and thin filaments (Figures 3A and 3B; Reedy, 1968). However, in contrast to the “double-chevron” arrangement of cross-bridges in an insect sarcomere, the cross-bridges in a vertebrate sarcomere show a higher variability regarding the distance between each other and tend to appear in clusters (Figures 3A, 3B, and 5). Although the most common composition of a cross-bridge is only one myosin double-head (Figure 3C), two consecutive myosin double-heads also appear at many target regions. Occasionally, there are also single heads bound to the thin filament (8.3% of all myosin heads) with their partners not identified within the tomogram. In addition, a rare “split-head” conformation also occurs when the two heads from one myosin binds to two different adjacent thin filaments (10 pairs observed; 0.6% of all heads) (Figures 3D and S4). This conformation has been previously suggested (Offer and Elliott, 1978) and vaguely indicated by 2D projection images (Hirose and Wakabayashi, 1993). Our observation provides direct proof of this conformation in three-dimensions in the rigor state vertebrate sarcomere. The ability of myosin to split its two heads and bind to different thin filaments enhances the plasticity of the sarcomere and allows proper contraction under slight structural deformation due to external forces.

**Figure 4:**
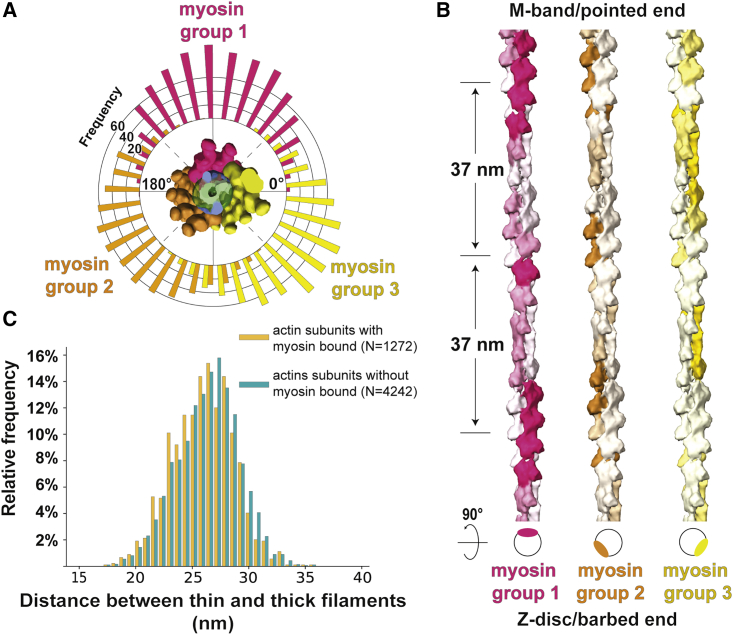
Myosin binding preference is dependent on actin orientation and distance between thin and thick filaments (A) Angular distribution of myosin heads shown by a circular histogram of the orientation of the actin subunits bound by myosin heads. The colors indicate three different thick filaments where the myosin heads originate. (B) Hotspots of myosin binding from three thick filaments shown on actin filaments. Actin filaments are colored with the footprint of the myosin heads according to multiple sequence alignment of the myosin binding profile sequences determined from the annotated volume and the fitted model in Figure 3B. See also Figure S5. The preferred side on a thin filament for binding of a certain myosin group is shown in the schematic diagram below. (C) Histograms of distances between thin and thick filaments at actin subunits with (orange) or without (green) a myosin head bound. Mean distances are 25.9 nm (SD 2.7 nm) and 26.3 nm (SD 2.7 nm), respectively. No skewness was measured in either population, meaning both distributions can be considered Gaussian.

**Figure 5:**
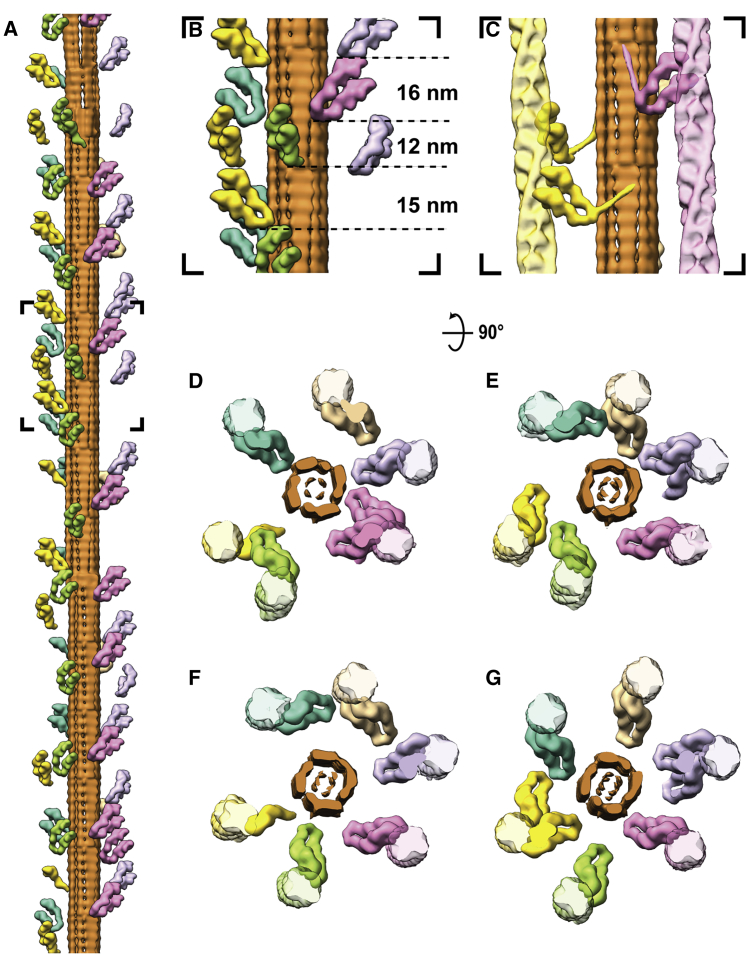
Arrangement of myosin heads around a thick filament (A) Model of a thick filament with surrounding thin filament-bound myosin heads obtained from fitting into the annotated tomogram. The six different colors represent myosin heads bound to the six adjacent thin filaments. (B) Close-up view of the black inset in (A) showing varying spacings between the tips of adjacent myosin heads. (C) Different conformations of S2 fragments taken from clearly discernible annotations. (D–G) Top view of four segments of the thick filament in (A) together with the thin filaments it binds, highlighting heterogeneity of myosin head organization. Each image depicts a segment with a depth of ~40 nm. See also Figures S2 and S4 and Video S1.

Notably, although we annotated mostly the C-zone of the A-band, where myosin binding protein C (MyBP-C) is present, we did not identify any corresponding densities along the thick filaments with a regularity of 43 nm as seen in previous studies (Craig and Offer, 1976; Luther et al., 2011). In the rigor state sarcomere, the density corresponding to MyBP-C is likely to be overwhelmed by the large number of myosin heads bridging between the thick and thin filaments. Further structural investigation on relaxed myofibrils will shed light on the MyBP-C organization as retracted myosin heads abolish the cross-bridges, leaving only cross-links formed by MyBP-C between the thick and thin filaments.

**Figure S5:**
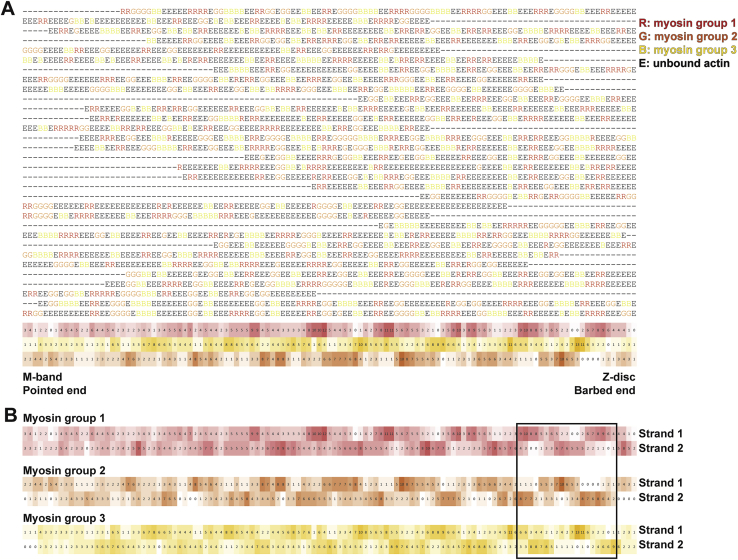
Overall myosin binding profile generated from multiple sequence alignment of myosin binding profiles of 30 thin filaments, related to Figure 4 and STAR Methods (A) The fitted myosin head model in Figure 3B was converted to myosin binding profile sequences on thin filaments, with “R,” “G,” “B” representing actin subunits bound by three groups of myosin heads originating from three neighboring thick filaments and “E” representing myosin-free actin subunits. These sequences were aligned using multiple sequence alignment with a customized weight matrix. Only the sequence of one actin strand for each thin filament was used for alignment. The sum of occurrence of each myosin group at each actin subunit position is shown at the bottom and colored accordingly. Darker color indicates higher occurrence. (B) The colored overall myosin binding profile on both actin strands for each myosin group. Sequences of strand 2 were combined using the alignment of strand 1 shown in (A). The overall myosin binding profiles depict hotspots for myosin binding on a thin filament and the region highlighted in the black box is used to color the models shown in Figure 4B.

To further investigate the variability in myosin binding, we focused on the influence of actin orientation and the distance between thin and thick filaments (Figure 4). We plotted the orientation of the actin subunits bound by myosin heads in terms of their relative angles with respect to a fixed orientation perpendicular to the filament axis. The distribution showed three distinctive groups with a Gaussian distribution corresponding to the three thick filaments from which the myosin heads originate. For a specific thick filament, the orientations of myosin-bound actin subunits are mostly confined within a range of ~120° (Figure 4A). This indicates that myosin heads tend to bind to actin subunits oriented toward the direction of the thick filament from which it originates. This is also demonstrated by the combined myosin binding profile of all 30 thin filaments (Figures 4B and S5). In addition, this footprint map of myosin binding on a thin filament also exhibits the 37-nm hotspot (~13 actin subunits) of cross-bridge clusters as a consequence of this preferred binding orientation of actin subunits.

Furthermore, the distances between the thin and thick filaments at both myosin-bound and myosin-free actin subunits show a Gaussian distribution with no obvious skewness (Figure 4C). The myosin-bound actin subunits are on average 0.4 nm closer to the thick filaments (95% confidence interval 0.2–0.5 nm) compared to myosin-free actin subunits. This small difference implies only a minor preference for myosin-binding when there is a shorter distance between the thin and thick filaments.

Our molecular map of myosin head distribution enabled us to determine the arrangement of myosin heads emanating from thick filaments within the rigor state sarcomere (Figure 5A). In the OFF state, when myosin heads are retracted to the thick filament, they are arranged in a 3-start helical pattern with 14.3 nm between layers of crowns as illustrated by cryo-EM and X-ray diffraction studies (Al-Khayat et al., 2013; Squire, 2009). This regular organization is not maintained in sarcomeres in the rigor state, when thick filaments are in the ON state. We could observe that the distance between axially adjacent myosin heads varies (Figure 5B) and found a different binding of myosin heads in different sections of a thick filament (Figures 5D–5G). The S2 fragment, which forms a convex surface with the S1 fragment (Cantino et al., 2000), provides enough flexibility for a myosin head to bind to a random actin subunit within the range of allowed distance and orientation (Figure 5C). This flexibility makes allowance for the mismatch of the 37-nm actin repeat in thin filaments and the 14.3-nm myosin repeat in thick filaments (Squire, 2009) and results in the pseudo-regular arrangement of cross-bridges.

Overall, the analysis of myosin heads in the A-band suggests that myosin binding is a stochastic process regulated by physical limitations such as the orientation of actin subunits. Myosin prefers to bind to regions on thin filaments that orient toward the thick filament, creating ~37 nm regular periodic cross-bridge clusters, consistent with previous observations in insect muscle (Reedy, 1968) and single-molecule experiments (Steffen et al., 2001). In insect flight muscle, cross-bridges are formed in distinct lead and rear bridges (Liu et al., 2004; Schmitz et al., 1996; Taylor et al., 1984). However, cross-bridges in vertebrate sarcomere are composed of various myosin double-heads in an irregular pattern. This pseudo-regular organization indicates a certain plasticity within a vertebrate sarcomere, whereby a strictly ordered myosin binding network is unnecessary.

### Thin filaments in the I-band are disorganized and bear a different tropomyosin state to those in the A-band

**Figure 6:**
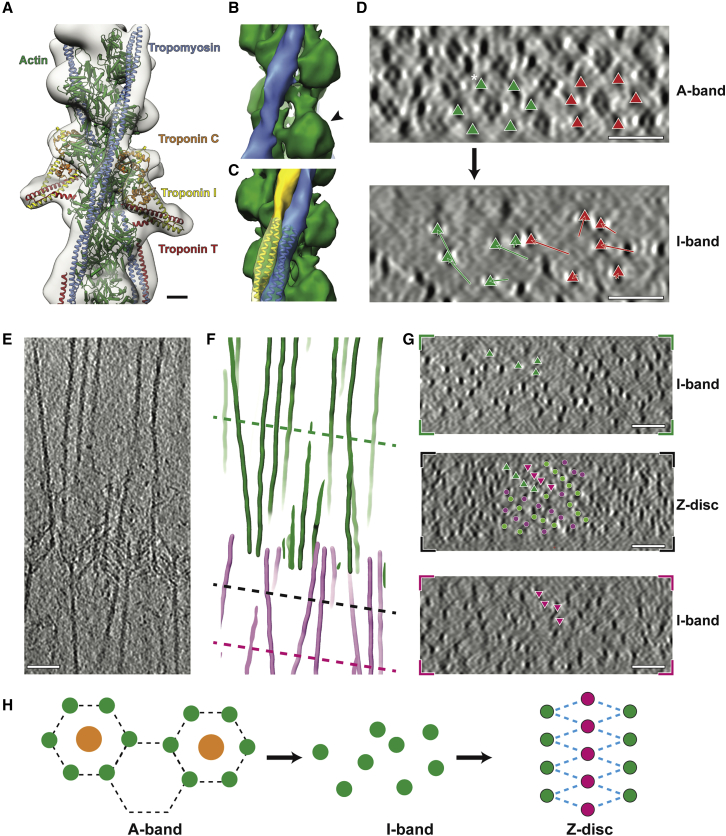
Structure and organization of thin filaments in the I-band (A) Sub-volume average of the thin filament in the I-band fitted with a homology model of the complete thin filament including troponin. Homology model is based on PDB: 6KN8. (B) 3D reconstruction of the thin filament in the I-band excluding troponin. The actin filament is depicted in green and tropomyosin in blue. At this resolution, the four subdomains of actin form a clear “U” shape (marked by the arrow head) and indicates actin barbed end faces toward the Z-disc. (C) Tropomyosin along thin filaments takes the C-state in the I-band (blue) and the M-state in the A-band (yellow). (D) XZ views of sarcomeres at the A-band and I-band highlighting the disappearance of the hexagonal pattern of thin filaments. Two hexagonal units of thin filaments and their corresponding positions in the I-band are indicated by green and orange triangles. Displacement of the filaments from A-band to I-band are shown as arrows. Asterisk denotes a filament that moved out of the field of view during the A–I transition. (E) Slice through a tomogram depicting the Z-disc and two I-bands from two adjacent sarcomeres. (F) 3D model of thin filaments showing the same region as in (E). (G) Cross-section views of the positions indicated by dotted lines in (F), showing the pattern of thin filaments during the I-Z-I transition. Filaments are traced in green triangles from the I-band to Z-disc of the top sarcomere and in magenta triangles from the Z-disc to the I-band of the bottom sarcomere. In the Z-disc image, antiparallel filaments in the center are labeled with green and magenta dots. (H) A schematic diagram shows the hexagonal pattern in the A-band, the irregular pattern in the I-band and the rhomboid pattern in the Z-disc. Scale bars, 2 nm (A), 50 nm (D–G). See also Figure S6.

One advantage of our approach is that we can trace single filaments through the sarcomere in 3D, allowing us to follow thin filaments from the A-band through the I-band into the Z-disc. The ordered hexagonal pattern of filaments in the A-band breaks down in the I-band, where the thick filaments end (Figure 6D). Consistent with previous studies presenting cross-section images of the I-band (Pepe, 1975) and contrary to a previous model stating an equally distanced arrangement of thin filaments along the A-band to Z-disc transition (Knupp et al., 2002), thin filaments show a transition in the I-band from a hexagonal pattern to an irregular pattern caused by the lack of cross-bridges in this region. The extent of displacement from the hexagonal arrangement varies among filaments (Figure 6D).

**Figure S6:**
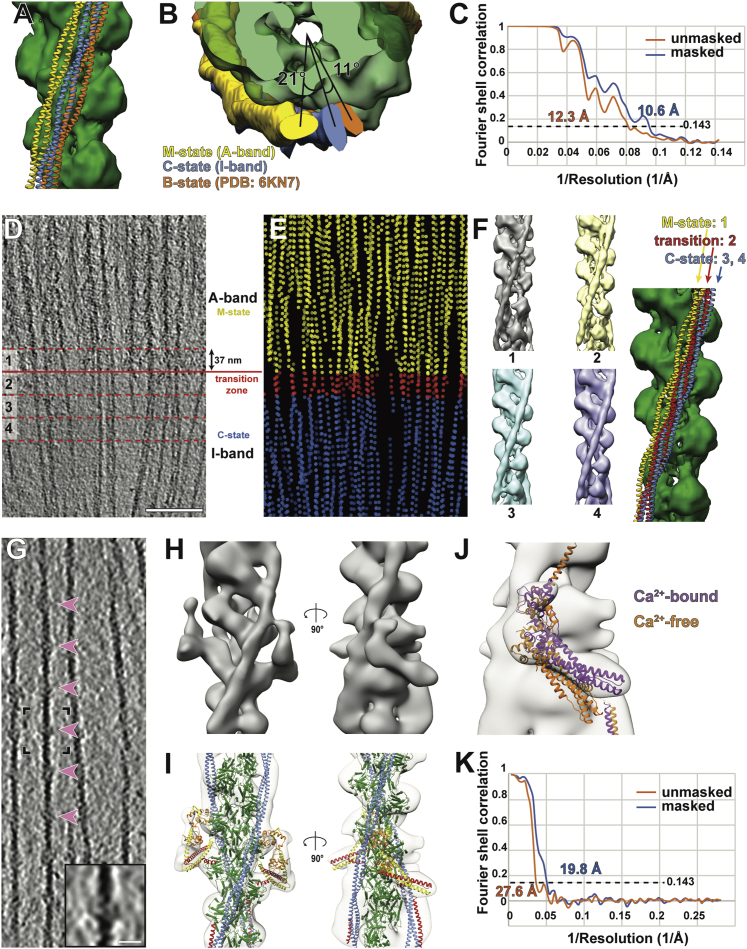
Sub-volume averaging of the thin filaments in the I-band, related to Figure 6 (A and B) Comparison of tropomyosin positions in different regions of the sarcomere. Side (A) and top (B) views of a thin filament with tropomyosin in the A-band (yellow) and I-band (blue). Tropomyosin is in the M state in the A-band and resides in the C-state in the I-band. The two states differ by an azimuthal rotation of ~21°. The B-state tropomyosin from a model of Ca^2+^-free thin filament (PDB: 6KN7) (orange) is also shown for comparison. (C) The estimated resolution of the reconstruction of a thin filament excluding troponin in the I-band is 10.6 Å using the 0.143 criterion. (D) Slice from a tomogram showing both A-band and I-band. The solid red line represents the border between the A-band and I-band. 37-nm sections separated by the red dotted lines were selected for separate sub-volume averaging. (E) 3D view of particles of the thin filaments in the same region as (D), colored based on the state of their tropomyosin position. All A-band particles exhibit the M-state tropomyosin position (yellow). Most I-band particles exhibit the C-state tropomyosin (blue). The particles in the closest 37-nm section in the I-band to the A-band (section 2 in (D)) exhibit an intermediate position of tropomyosin, and hence define a transition zone. (F) Averaged structures corresponding to sections 1-4 as indicated in (D). The structures were aligned to a non-decorated F-actin structure. Tropomyosin models fitted into the structures are shown on the right. Section 1 has the same tropomyosin position as the M-state structure. Section 3 and 4 has the same tropomyosin position as the C-state structure. Section 2 has an intermediate tropomyosin position between the M- and C-state, implying that this is the section where the transition occurs. (G) Slice of a tomogram depicting thin filaments in the I-band. Bulges along the filaments appear with a periodicity of ~37 nm (highlighted by pink arrow heads), corresponding to troponin complexes. An enlarged version of a pair of troponin complexes in the black inset is shown at the bottom-right corner, depicting clear extra densities. Scale bar, 10 nm. (H) 3D reconstruction of a thin filament decorated by a pair of troponin complexes obtained from sub-volume averaging. (I) A homology model based on the structure of the Ca^2+^-bound cardiac muscle thin filament (PDB: 6KN8) was fitted into the map. Actin, tropomyosin, troponin I, troponin T and troponin C are shown in green, blue, yellow, red, and orange, respectively. (J) The structure of troponin in the Ca^2+^-bound state (PDB: 6KN8) fits much better into the map than the structure of troponin in the Ca^2+^-free state (PDB: 6KN7). Together with the C-state position of tropomyosin (A and B), this indicates that the I-band thin filament is in the Ca^2+^-bound state. (K) The estimated resolution of the reconstruction of the thin filament including troponin in the I-band is 19.8 Å using the 0.143 criterion.

Due to the absence of myosin in the I-band, troponin complexes are clearly visible with a periodicity of ~37 nm on the thin filament (Figures 1C, 6D, and S6). We applied sub-volume averaging to determine the structures of the actin-tropomyosin-troponin complex and the actin-tropomyosin complex in the I-band at resolutions of 19.8 Å and 10.6 Å (Figures 6A, 6B, and S6), respectively. We then calculated a homology model using an *in vitro* cardiac muscle thin filament cryo-EM structure of the Ca^2+^-bound state as a template (PDB: 6KN8) (Yamada et al., 2020) and fitted it into the density maps using rigid body fitting. The model fits well into the reconstruction of both structures, demonstrating that the thin filament is in the on-state with tropomyosin in the C (Ca^2+^ induced) state position (Figures S6I and S6J). This suggests that there is structural similarity between skeletal and cardiac troponin complexes when bound to actin filaments at this resolution, despite differences in respective crystal structures (Takeda et al., 2003; Vinogradova et al., 2005). Interestingly, the position of tropomyosin differs from that in the A-band, where tropomyosin is in the M (myosin-bound) state (Figures 6C, S6A, and S6B; von der Ecken et al., 2016; McKillop and Geeves, 1993). Thus, the binding of myosin to the thin filament shifts tropomyosin from the C-state to the M-state position in the A-band, whereas tropomyosin remains in the C-state in the I-band. Although different tropomyosin states have been observed separately *in vitro* (von der Ecken et al., 2015, 2016; Poole et al., 2006; Yamada et al., 2020), our *in situ* structures of thin filaments suggest that the tropomyosin position can vary on a local scale within the same sarcomere and even the same thin filament.

To determine the location of this transition, we subdivided the tomograms in 37-nm sections around the A-band/I-band transition and averaged the thin filaments separately (Figure S6D). The averages revealed that tropomyosin is in the M-state in A-band sections. In the first I-band section, tropomyosin is in an intermediate state between the M-state and C-state and from the second I-band section, it occupies the C-state position (Figure S6F). This suggests that, contrary to previous predictions, the M- to C-state transition happens mostly within one tropomyosin unit and occurs at the section immediately after the A-band/I-band transition.

### Different forms of the Z-disc and the organization of α-actinin network

**Figure 7:**
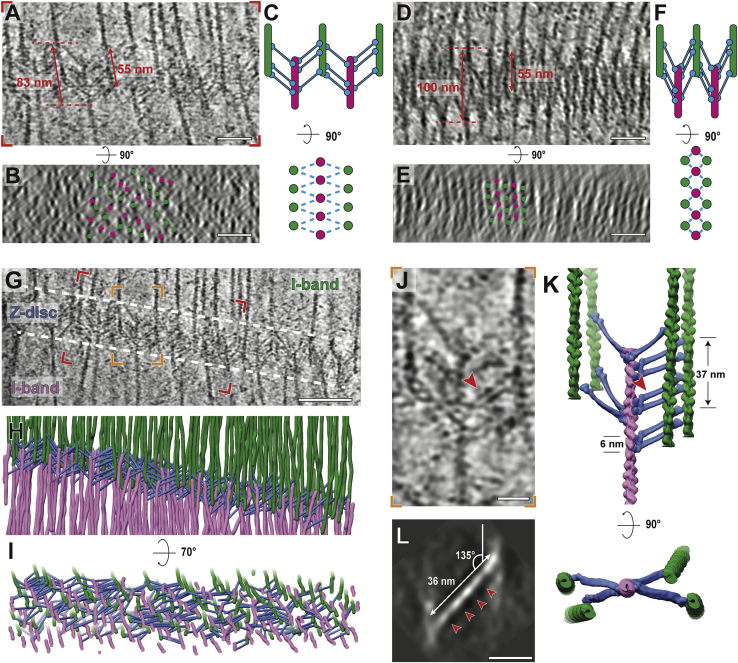
Different types of Z-discs from fast mouse psoas myofibril and α-actinin organization in the thinner form (A–C) XY slice view (A), equator-filtered cross-section view (B), and a schematic diagram showing the pattern (C) of a Z-disc in the thinner form within a tomogram. The thickness of the Z-disc was measured between where α-actinins bind to the antiparallel thin filaments. The length of a single thin filament within the Z-disc was also measured. The organization of the filaments in this Z-disc is rhomboidal, likely resulting from a tilted orientation of the Z-disc on the grid and a slight stretching of the myofibril. We used this Z-disc for our analysis because most of the α-actinin densities could be unambiguously assigned. Scale bar, 50 nm. (D–F) XY slice view (D), cross-section view (E), and a schematic diagram of a tomogram showing a Z-disc in the thick form with a square pattern (F). This is the predicted organization of a Z-disc from skeletal muscle. However, it is difficult to assign individual α-actinin densities in these tomograms hampering a non-biased annotation. Scale bar, 50 nm. (G) Slice through the same tomogram as (A) depicting the thin-form Z-disc and two I-bands. Scale bar, 100 nm. (H) Cryo-ET based 3D model of the Z-disc showing antiparallel thin filaments from two adjacent sarcomeres (green and magenta) and the α-actinins (blue) cross-linking the filaments. (I) Tilted view of the model shown in (H). (J) Slice of an example unit in the Z-disc depicting one thin filament with its neighboring antiparallel thin filaments and the α-actinins connecting them. A doublet of α-actinins is highlighted by a red arrow head. Scale bar, 20 nm. (K) Cryo-ET-based 3D model of the same region in (J). Actin filament model is derived from the thin filament reconstruction in Figure 6A, excluding tropomyosin. The α-actinin model is derived from the crystal structure of an α-actinin dimer (PDB: 4D1E). (L) Projection image (7-nm thickness) of the 3D reconstruction of α-actinin obtained from averaging the sub-volumes as picked in (H) and (I), depicting four domains (marked by red arrow heads) which correspond to the four spectrin-like repeats (SRs) in the rod region. Scale bar, 20 nm. See also Figure S7 and Video S2.

**Figure S7:**
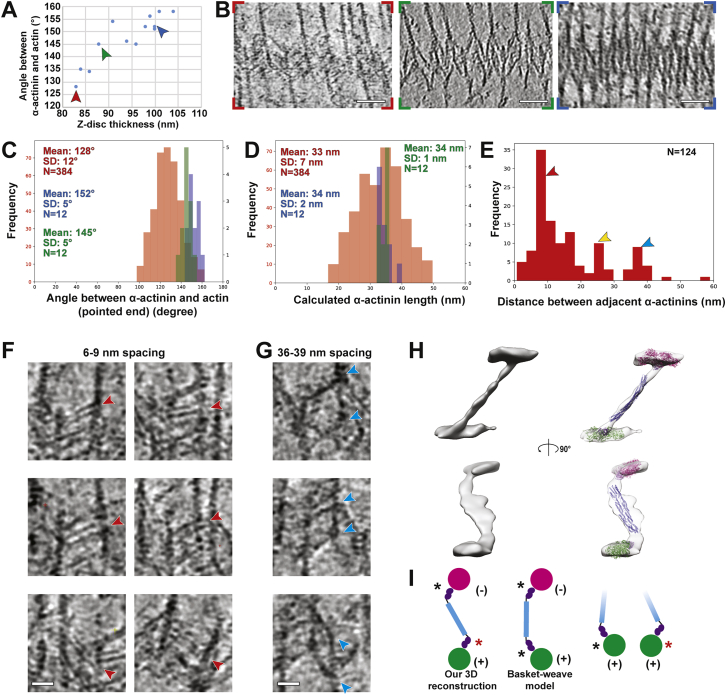
α-Actinin structure and organization in the Z-disc, related to Figure 7 (A) Plot relating the thickness of Z-discs and the angle between α-actinins and the pointed end of actin filament from different tomograms. The positive correlation implies a parallel hinge mechanism of the Z-disc. (B) Slices through tomograms depicting a thin Z-disc (left, red arrow in (A)), a thick Z-disc (right, blue arrow in (A)) and a Z-disc of intermediate thickness (middle, green arrow in (A)). Scale bar, 50 nm. (C) Distribution of the calculated angles between annotated α-actinins and actin filaments in direction to the pointed end in the thin (red), thick (blue) and intermediate-thickness (green) Z-discs. The y axis for both the thick and intermediate Z-disc is shown on the right in black. There are more data points for the thin Z-disc as it was completely annotated, while a few examples of α-actinin were selected for the intermediate and thick Z-discs. (D) Distribution of the length of annotated α-actinins in the thin (red), thick (blue) and intermediate-thickness (green) Z-discs. The distance along α-actinin between the centers of the connecting actin filaments was measured and the length of α-actinin was calculated by subtracting the diameter of an actin filament (6 nm). The relatively large standard deviation in the thin form Z-disc is likely caused by α-actinins binding to actin filaments at different azimuthal orientations and the error in the precise determination of the central axis of actin filaments. (E) Distribution of the calculated spacing between adjacent α-actinins in the thin Z-disc. The red, yellow, and blue arrow heads highlight peaks at 6-9 nm, 24-27 nm and 36-39 nm, respectively. The 24-27 nm peak appears as a result of the two other types of spacing (36 - 2x6). (F and G) Example images showing α-actinins with the 6-9 nm spacing (D) and the 36-39 nm spacing (E). Arrow heads highlight the positions and orientations of α-actinins. Scale bar, 20 nm. (H) 3D reconstruction of α-actinin obtained from sub-volume averaging. Although there is a strong missing wedge effect resulting in an elongation of the reconstruction in one direction, we were able to manually fit atomic models derived from the crystal structure of α-actinin (PDB: 4D1E) and the cryo-EM structure of actin filaments bound by the first calponin homology domain of the actin binding domain (PDB: 6D8C) into the density. Actin filaments are shown in magenta and green; the actin binding domain and the rod region are depicted in purple and blue, respectively. The flexible neck regions and the C-terminal calmodulin-like domains are not shown. (I) Left: Schematic diagram showing the difference between averaged α-actinin structure and the conventional basket-weave model. Right: The two different interactions (marked by the red and black asterisks) between the end of α-actinin and actin filaments (magenta and green) are demonstrated on the right, formed by the flexible neck region between the rod (blue) and actin binding domain (purple).

The irregular pattern of thin filaments resumes an ordered state when the filaments approach the Z-disc (Figure 6G). In contrast to the exact square patterns observed in Z-discs from midshipman fish sonic muscle (Burgoyne et al., 2019) and rat soleus and cardiac muscle (Goldstein et al., 1990), the Z-discs in our reconstructions are slightly less well ordered and form squared to more rhomboid patterns (Figures 6G, 6H, and 7). This is likely due to the fact that we used single myofibrils instead of complete muscles, where Z-discs are laterally anchored and stabilized. Antiparallel thin filaments in the Z-disc with opposing polarities are connected by 33-nm long cross-links, which we attribute to α-actinin (Ribeiro et al., 2014; Takahashi and Hattori, 1989; Figures 7G–7I and S7D).

By comparing Z-discs from different reconstructions, we found different Z-discs varied in thickness, ranging from ~80 nm to ~100 nm (Figures 7A–7F, S7A, and S7B; STAR Methods). Thicker Z-discs are more compact than thinner ones (thin filaments are closer laterally) (Figures 7A–7F and S7B). Interestingly, although the mean length of α-actinin is similar in all Z-discs (Figure S7D), the average angle between α-actinin and actin filaments in direction to the pointed end differs considerably, ranging from ~158° in the thickest and ~128° in the thinnest form (Figure S7A).

We propose that the different forms of Z-discs represent conformational states of the sarcomeric unit. We hypothesize that post-isolation mechanical factors, such as different on-grid blotting forces before freezing, increase the stress/strain in some myofibrils. The angle between actin and α-actinin becomes more acute and the Z-discs fold together like a parallel hinge, resulting in the thicker appearance of the Z-disc (Figures 7D–7F, S7A, and S7B). This situation is likely similar to activated myofibrils under tension (Pavlov et al., 2009). Thinner forms possibly represent the Z-disc under low strain/stress, where the angle between actin and α-actinin is more obtuse (Figures 7A–7C, S7A, and S7B). Considering a stable interaction interface between the actin-binding domain of α-actinin and actin filaments (Iwamoto et al., 2018) and the rigidity of the rod region of α-actinin (Ribeiro et al., 2014), the flexibility in the neck between the rod and the actin-binding domain (ABD) of an α-actinin probably serves as the central hinge of this pivot-and-rod structure (Gautel and Djinović-Carugo, 2016). In order to experimentally validate that stress/strain induces different conformations of the Z-disc, activated myofibers will have to be vitrified under defined strain/stress conditions prior to cryo-ET analysis in the future.

We annotated all clearly visible densities in the thinner Z-disc where single α-actinin densities were more apparent to understand the α-actinin organization between antiparallel thin filaments (Figure 7G; Video S2). Top and side views of the resulting three-dimensional model reveal that α-actinin forms a mesh by cross-linking the actin filaments, which is less well ordered than anticipated from the projection (Figures 7G–7I) or from idealized helical models in previous work (Luther, 2009). A prominent and unexpected feature is a doublet of α-actinin cross-links with 6 nm spacing, which is only possible when two α-actinins bind to longitudinally adjacent actin subunits along the actin filament (Figures 7J, 7K, and S7F). We observe up to three of these α-actinin pairs connecting two antiparallel thin filaments over ~37 nm, at ~18.5-nm intervals. Thus, although the spacing of these doublets follows the 37-nm actin helical repeat, it places individual α-actinin molecules closer together (maximally 12 nm rather than 18.5 nm) than previously presumed. Doublets have also been observed in an *in vitro* reconstituted α-actinin-F-actin raft (Hampton et al., 2007). Our analysis shows that this arrangement was not an *in vitro* artifact but a feature of native sarcomeres. Interestingly, these doublets were not apparent in the recent reconstruction of the F-actin-α-actinin complex from branched cardiac myofibrils obtained through sub-volume averaging, in which the angles between α-actinin and actin resemble the thick-form Z-disc (Oda and Yanagisawa, 2020). Importantly, as single α-actinins were not distinguishable in these tomograms, this study relied strongly on subtomogram averaging at certain positions, assuming a highly symmetric Z-disc. However, our visualization of the Z-disc illustrates that it is a heterogeneous, non-symmetrical structure. The doublets, which are not present at regular intervals, were therefore probably lost during sub-volume averaging of the Z-disc or are not present in cardiac myofibrils.

Video S2. Molecular organization of the Z-disc, related to Figure 7 Slices of a tomogram depicting the Z-disc of a sarcomere are shown by moving through the Z axis. The traced thin filaments (green and magenta colors for filaments with opposing polarity) and annotated α-actinins (blue) are modeled as cylindric volumes and shown in a 3D space.

Consistent with previous studies (Burgoyne et al., 2019; Luther and Squire, 2002), we observed several ~37 nm spaced α-actinin cross-links (Figures S7E and S7G), which corresponds roughly to the half-helical pitch of actin filaments. However, we also observed α-actinins that do not follow this pattern (Figures 7J, 7K, and S7E) suggesting a less ordered organization of the Z-disc than previously assumed or inferred from atypical actin-α-actinin arrays in nemaline rods or midshipman muscle (Burgoyne et al., 2019; Morris et al., 1990). By performing sub-volume averaging of the α-actinin positions, we could show that it connects opposite sides of antiparallel actin filaments, forming a slight “S” shape (Figure S7H). The actin binding domains and the central rod region of an α-actinin from the crystal structure (Ribeiro et al., 2014) fit well into the density (Figure S7H), and the four spectrin-like repeats can be clearly distinguished in a projection of the reconstruction (Figure 7L). This “S” shape fits well to the α-actinin crystal structure (Ribeiro et al., 2014), considering the flexibility in the neck region, but differs from the conventional “basket-weave” model that is derived from observations of transverse sections of Z-discs (Ullrick et al., 1977). In order to form the S shape, the two ends of an α-actinin, where it interacts with actin filaments, need to have different conformations; one end resembling that of the basket-weave model with the other end in an alternate conformation that interacts with actin differently at the flexible neck region (Figure S7I). Future studies at a higher resolution will elucidate the molecular basis of this interaction and shed light on the organization of other proteins in the Z-disc, such as accessory smaller proteins like ZASP, myotilin or FATZ that may play a role in α -actinin dimerization, or to visualize the position of the α-actinin-binding Z-repeats of titin or the actin-binding repeats of nebulin. The potential contribution of these proteins to the arrangement of α-actinin doublets would also be of fundamental interest for understanding diseases of the Z-disc with aberrant arrangement of alpha-actinin like actininopathies (Savarese et al., 2019), zaspopathies (Griggs et al., 2007), nemaline myopathies (Wallgren-Pettersson et al., 2011), or myofibrillar myopathies (Selcen and Engel, 2011).

## Conclusions

Cryo-FIB milling combined with cryo-ET enabled us to visualize native vertebrate sarcomeres in three-dimensions with minimal artifacts or damage. Our structure of the actomyosin complex shows the complete myosin double head in the rigor state in molecular detail and pinpoints the ELC/RLC interface as the position within myosin crucial for the structural rearrangement of the double head when bound to the thin filament. In addition, our model of the sarcomere shows a pseudo-regular distribution of myosin cross-bridges in the A-band. An irregular pattern of the I-band thin filaments illustrates the importance of the cross-bridge and the Z-disc in maintaining the regularity of thin filaments within the sarcomere. Our observation that the thin filaments and cross-links of α-actinin within the Z-disc can accommodate different arrangements demonstrates the need for the sarcomere to respond to and accommodate the collective forces exerted on it during muscle contraction. Collectively, our high-resolution structure of the sarcomere, although not yet a complete atomic model of the entire structure, identifies previously unresolved details of the molecular architecture of A-bands, I-bands, and Z-discs and highlights the molecular plasticity of its components. It allows improved dynamic modeling of muscle contraction and will serve to test new concepts on the molecular, cellular, and physiological level, not least for the development of new approaches for investigating muscle diseases.

### Limitations of the study

Although this study provided an overall picture of the skeletal sarcomere, subsequent work needs to be performed to address a number of missing pieces arising from this research. In this study, we focused on sarcomeres from skeletal muscle in rigor, where the myosin cross-bridges are locked in a strong-binding state. Other states of the cross-bridge cycle need to be investigated in order to provide a dynamic understanding of muscle contraction. This study could not include an analysis of M-band proteins, MyBP-C, or other less regularly arranged proteins that also play an essential role in sarcomere regulation, function, and plasticity. Additional *in situ* labeling methods in future studies will help to elucidate the organization of these less common and less organized proteins. In the analysis of Z-discs of different thicknesses, our hypothesis that the different Z-disc conformations may correspond to the different stain/stress states of the sarcomere should be validated experimentally using custom approaches where muscle can be examined under defined load. Future studies using greater numbers of tomograms acquired on myofibrils should help to improve the resolution of structures obtained in this study to achieve insights on the atomic level.

## STAR★Methods

### Key resources table

**Table:** 

REAGENT or RESOURCE	SOURCE	IDENTIFIER
**Biological samples**		

Mouse fast psoas myofibrils from a 3-month female BALB/c mouse	King’s College London	N/A

**Deposited data**		

Sub-volume average of *in situ* actomyosin complex	This paper	EMD: 12289
Sub-volume average of re-centered myosin double-head	This paper	EMD: 12291
Sub-volume average of I-band thin filaments without troponin	This paper	EMD: 12292
Sub-volume average of I-band thin filaments including troponin	This paper	EMD: 12293
Homology model of *in situ* actomyosin complex	This paper	PDB: 7NEP
Cryo-EM structure of *in vitro* cytoplasmic actomyosin complex	von der Ecken et al., 2016	PDB: 5JLH
Crystal structure of rigor-like squid myosin S1 fragment	Yang et al., 2007	PDB: 3I5G
Cryo-EM structure of human cardiac thin filament in calcium-bound state	Yamada et al., 2020	PDB: 6KN8
Cryo-EM structure of human cardiac thin filament in calcium-free state	Yamada et al., 2020	PDB: 6KN7
Cryo-EM structure of relaxed myosin heads in insect flight muscle	Hu et al., 2016; Knupp et al., 2019	PDB: 6SO3
Crystal structure of human myosin S2 fragment	Blankenfeldt et al., 2006	PDB: 2FXM
Crystal structure of human α-actinin 2	Ribeiro et al., 2014	PDB: 4D1E
Cryo-EM structure of actin filament decorated by filamin A CH1 domain	Iwamoto et al., 2018	PDB: 6D8C

**Software and algorithms**		

SerialEM	Mastronarde, 2005	https://bio3d.colorado.edu/SerialEM/
MotionCorr2	Zheng et al., 2017	https://emcore.ucsf.edu/ucsf-software
EMAN2	Tang et al., 2007	https://blake.bcm.edu/emanwiki/EMAN2
IMOD	Kremer et al., 1996	https://bio3d.colorado.edu/imod/
crYOLO	Wagner et al., 2019	https://cryolo.readthedocs.io
RELION3	Bharat and Scheres, 2016	https://www3.mrc-lmb.cam.ac.uk/relion/index.php?title=Main_Page
PEET	Heumann et al., 2011; Nicastro et al., 2006	https://bio3d.colorado.edu/PEET/
ISAC	Yang et al., 2012	http://sphire.mpg.de/wiki/doku.php?id=gpu_isac
SPHIRE	Moriya et al., 2017	https://sphire.mpg.de/
TrackMate plug-in in Fiji	Tinevez et al., 2017	https://imagej.net/TrackMate
Fiji (ImageJ)	Schindelin et al., 2012; Schneider et al., 2012	https://imagej.net/Fiji
TEMPy	Farabella et al., 2015	http://tempy.ismb.lon.ac.uk/
SWISS-MODEL	Bertoni et al., 2017; Bienert et al., 2017; Guex et al., 2009; Studer et al., 2020; Waterhouse et al., 2018	https://swissmodel.expasy.org/
Chimera	Pettersen et al., 2004	https://www.cgl.ucsf.edu/chimera/
Amira2019.3	Thermo Fisher Scientific	https://www.thermofisher.com/us/en/home/industrial/electron-microscopy/electron-microscopy-instruments-workflow-solutions/3d-visualization-analysis-software/amira-life-sciences-biomedical.html
MUSCLE algorithm in msa R package	Edgar, 2004	https://www.rdocumentation.org/packages/msa

### Resource availability

#### Lead contact

Further information and requests for resources and reagents should be directed to and will be fulfilled by the Lead Contact, Stefan Raunser (stefan.raunser@mpi-dortmund.mpg.de).

#### Materials availability

This study did not generate new unique reagents.

#### Data and code availability

The EM structures generated through this study have been deposited in the Electron Microscopy Data Bank (https://www.ebi.ac.uk/pdbe/emdb) under accession numbers 12289, 12291, 12292, 12293. The *in situ* actomyosin model is deposited in the Protein Data Bank (https://www.rcsb.org) under accession number 7NEP.

### Experimental model and subject details

This study used muscle isolated from the psoas of 3-month female BALB/c mice. Animals were euthanised in a schedule-1 procedure by cervical dislocation following licensed procedures approved by King’s College London ethics committee and the Home Office UK. The isolation of muscle is described below in the Method details.

### Method details

#### Myofibril preparation and vitrification

Myofibrils were isolated from BALB/c mouse psoas muscle essentially as previously described (Knight and Trinick, 1982; Shalabi et al., 2017). Briefly, bundles of the freshly excised *psoas major* muscle were tied to plastic supports and their sarcomere length adjusted as judged by laser diffraction to 2.2 - 2.4 μm. They were allowed to equilibrate in rigor buffer (20 mM HEPES pH 7, 140 mM KCl, 2 mM MgCl_2_, 1 mM EGTA, 1 mM DTT, Roche complete protease inhibitor) over night at 4°C. The following day, the central section of the bundles was dissected into ~2 mm pieces and homogenized 3 to 4 times at 7,700 rpm for 15 s, and 3 times at 10,000 rpm for 5 s (IKA TC10 basic ULTRA-TURRAX® homogenizer with S10N-5G dispersing element, IKA England) with intermittent washes in rigor buffer, with myofibril separation monitored by light microscopy of small samples at each step. Myofibril suspensions were stored in complete rigor buffer at 0°C for 1-3 days until vitrification. Grids with myofibrils were vitrified using a Vitrobot Mark IV plunger (Thermo Fisher Scientific, USA). 2 μl of myofibril-containing solution was applied to glow-discharged Quantifoil R1.2/1.3 Cu 200 grids. After incubation on the grid for 60 s at 13°C, excess solution was blotted for 15 s from the opposite side from the sample with a Teflon sheet attached to the front pad of the apparatus. The grids were then vitrified by plunge-freezing into liquid ethane and afterward clipped into cryo-FIB-specific autogrid rings (Thermo Fisher Scientific) with marks for orientation alignment and a cut-out for subsequent milling at a shallow angle.

#### Cryo-focused ion beam milling

Clipped autogrids were loaded into a shuttle and transferred into an Aquilos cryo-FIB/SEM dual-beam microscope (Thermo Fisher Scientific). Overview tile sets for the grids were acquired at 256 x magnification with the scanning electron beam using MAPS software (Thermo Fisher Scientific). The grids were then sputter coated with platinum for 15 s to minimize charging effects arising from the electron beam, allowing better recognition of myofibrils (Figure 1). Vitrified myofibrils were localized and labeled as lamella sites. Prior to FIB-milling, organometallic platinum was deposited onto the grids through a gas-injection-system to prevent damage to the sample at the leading edge from the gallium ion beam. For each lamella site, the coincident point between electron beam and ion beam was determined by adjusting stage Z height. The stage was tilted to allow a 6-10° incidence angle of ion beam. FIB-milling using gallium ions was then performed in four steps as described in Table S1. During each step, all lamellae were milled before proceeding to the next step. All lamellae were polished within one hour to minimize water deposition onto the surface of lamellae. During polishing, the lamella was monitored with the electron beam at 5 kV, 25 pA to help estimate its actual thickness via charging propensity.

#### Electron cryo-tomography and tomogram reconstruction

Autogrids were rotated by 90° after milling before being inserted into an autogrid cassette so as to align the longitudinal axis of lamellae perpendicular to the tilt axis of the transmission electron microscope (TEM) stage later. This ensures autofocus and record positions are aligned along the tilt axis on a lamella and have close to the same eucentric height. Grids were then loaded into a Titan Krios (Thermo Fisher Scientific) equipped with a zero-loss energy filter and a K2 Summit direct electron detector (Gatan Inc., USA). SerialEM software (Mastronarde, 2005) was used to acquire images. Lamellae overview images were acquired at 6,300x or 8,400x magnification. High magnification tilt series were acquired at a calibrated post-energy-filter TEM magnification of 28409x (nominal magnification of 81,000x, pixel size 1.76 Å) with a dose-symmetric tilt scheme (Hagen et al., 2017) and a total dose ranging from approximately 130 to 155 e^-^/Å^2^. The stage was tilted from −60° to +60° relative to the lamella tilt angle (6 - 10°) with an increment of 3° during data acquisition. The defocus at which acquisition was performed ranged from 2.4 μm to 4 μm (Table S2). Collected tilt movies were subsequently motion-corrected and stacked in batch using a custom python script with MotionCorr2 (Zheng et al., 2017) and EMAN2 (Tang et al., 2007). Subsequent tomogram reconstruction steps including tilt series alignment, CTF estimation and correction, and weighted back projection were performed using the IMOD software package (Kremer et al., 1996). Platinum particles deposited onto the surface of lamellae were used as fiducial markers for alignment of tilt series where possible whereas in their absence patch tracking was used to align the tilt series.

#### Sub-volume averaging of the thick filament

Tomograms were initially 4x binned and lowpass-filtered to 60 Å using EMAN2 for visualization. To pick filaments accurately without the interference from signals from cross-bridges, an equatorial mask in the Fourier transform of XY slices of tomograms was applied. CrYOLO (Wagner et al., 2019) was afterward employed to detect the shape of thick filaments in the XZ planes of the filtered tomogram. The detected points were then traced by a python script, which generated the coordinates for the thick filaments. In total, 13,700 segments of thick filaments (sub-volumes) were picked from 8 tomograms from multiple myofibrils with an inter-segment distance of 105 Å. These segments were extracted from unbinned and 2x binned tomograms with a box size of 128 pixels (450 Å) using RELION (Bharat and Scheres, 2016). Prior information of the orientations of the thick filaments in a tomogram was used to generate a featureless cylinder-like reference using PEET (Heumann et al., 2011; Nicastro et al., 2006). The sub-volumes were then aligned and averaged using RELION. Three different strategies were used during alignment. First, the sub-volumes were aligned without any symmetry imposed and a global refinement was enabled. This resulted in aligning the missing wedge and a map with anisotropic resolution was generated (Figure S2B). Then, a C3 symmetry was imposed during refinement according to a previous structural study of the relaxed human cardiac myosin filament (Al-Khayat et al., 2013). Although the averaged map had an isotropic resolution, the quality of the map did not improve (Figure S2C). In the end, without any symmetry imposed, only local refinement was enabled to prevent aligning the missing wedges (Figure S2D). This averaged structure has an estimated resolution of 30.4 Å based on the “gold-standard” FSC with 0.143 criterion. The map was used as a model for the thick filament in Figures 3 and 5.

#### Sub-volume averaging of the actomyosin complex and fitting of atomic model

Thin filaments were picked automatically in a similar method as described above. Instead of using crYOLO, thin filaments, which appeared as dense dots from the XZ view (Figure S1E), were recognized and traced by the TrackMate plug-in (Tinevez et al., 2017) in Fiji (Schindelin et al., 2012; Schneider et al., 2012). In total, 32,421 segments (sub-volumes) were picked from 8 tomograms from multiple myofibrils with an inter-segment distance of 63 Å. These segments were extracted from unbinned and unfiltered tomograms with a box size of 200 pixels (351 Å) using RELION (Bharat and Scheres, 2016). In order to exclude low-quality sub-volumes using 2D classification, the extracted sub-volumes were first rotated to orient thin filaments parallel the XY plane based on filament orientations calculated from particle positions. Projection images along Z axis were then generated from the central 100 slices (175 Å) of each particle and subsequently classified using 2D classification in the ISAC software (Yang et al., 2012) from the SPHIRE package (Moriya et al., 2017). This approach, compared to using the entire sub-volume for projection, excluded signals from other filaments at the corner of the sub-volume and thus enabled a more reliable assessment of particle quality (Figure S3B). 21,130 selected good sub-volumes were aligned, averaged, and classified in 3D using RELION, based on a cylinder-like reference generated from averaging all sub-volumes after aligning the longitudinal axis of segments using PEET (Figure S3C). In each 3D class, translation and rotation parameters were modified to align the most prominent double-head of myosin (Figure S3D). After combining the modified good classes and removing duplicate particles, 18,090 sub-volumes were refined locally with a mask including the thin filament and one pair of myosin double-head. The final average has an estimated resolution of 10.2 Å based on the “gold-standard” FSC with 0.143 criterion. Local resolution was estimated in SPHIRE using the two half-maps and the mask used during averaging. The final map was sharpened with a B-factor of −250 and filtered to the nominal resolution (Figure S3F).

In order to resolve the light chain domains of myosin heads, sub-volumes centered on myosin the double-heads (shifted 90 pixels along x axis compared to original sub-volumes) were extracted. The positions of these new sub-volumes in tomograms were calculated by a python script using the original sub-volume coordinates and their alignment parameters. The new sub-volumes were sorted through 3D classification in RELION. 4,519 particles from the two good classes were refined locally with masking out the thin filament and myosin double-head (Figures S3E and S3G).

An initial atomic model of the *in situ* actomyosin complex was first built by rigid-body fitting of actin subunits and myosin motor domains from the atomic model of the *in vitro* actomyosin complex (von der Ecken et al., 2016) (PDB: 5JLH), tropomyosin from the atomic model of isolated cardiac thin filament (Yamada et al., 2020) (PDB: 6KN8) and myosin lever arms from the crystal structure of rigor-like squid myosin S1 (Yang et al., 2007) (PDB: 3I5G) using ‘Fit in Map’ in Chimera (Pettersen et al., 2004). Essential light chains (ELCs) and the regulatory light chain (RLCs) were fitted separately into the averaged map after removing 5 amino acids (M796-Y800) at the hinge on the α-helix between ELC and RLC. The model of RLC together with part of the heavy chain lever arm (K801-L839) was rigid-body fitted into a segmented map generated by “Color Zone” in Chimera. Based on this initial atomic model and the sequences of actin, myosin and tropomyosin in mouse fast skeletal muscle, a homology model was calculated using SWISS-MODEL (Bertoni et al., 2017; Bienert et al., 2017; Guex et al., 2009; Studer et al., 2020; Waterhouse et al., 2018) (Figure 2). Comparison of lever arms in the *in situ* rigor upper head, lower head, squid myosin S1 fragment, and the blocked and free heads from relaxed insect flight muscle (PDB: 6SO3) (Knupp et al., 2019) (Figures S3I and S3J) was performed by aligning the RLC of the heads using the “MatchMaker” function in Chimera.

#### Annotation of the A-band and fitting of the molecular model of actomyosin

In order to visualize the organization of cross-bridges between thin and thick filaments, the densities of a complete A-band were annotated manually using Amira (Thermo Fischer Scientific) on a slice-by-slice basis. A user-defined mask based on gray values was used during manual annotation to prevent the picking of weaker densities. The 2D annotation stacked to form a 3D segmented volume depicting the arrangement of thin and thick filaments as well as the cross-bridges formed by myosin heads (Figure 3A). During sub-volume averaging, a structure of the thin filament fully decorated with myosin heads (Figure S3C) was obtained prior to 3D classification. This was low-pass filtered to 17 Å and extended to a length of 1,755 Å (~63 actin subunits) based on the helical symmetry detected in the structure (−166.6° turn and 27.9 Å rise) using RELION helix toolbox. This long fully-decorated thin filament model was then fitted into each segmented volume corresponding to a thin filament with bound myosin heads using “Fit in Map” in Chimera. The myosin heads that matched segmented volume were kept and colored depending on which thick filament they originated from while the other myosin heads were removed. This generated a complete map of myosin heads bound to thin filaments in the A-band (Figures 3B and 5; Video S1).

The models of double-head and split-head conformations were made from initially fitting our actomyosin structure into the segmented volume (Figures 3C, 3D, and S4). For the split-head conformation, the upper head from the double-head conformation was fitted into densities for both heads. The S2 domain from segments of a crystal structure (Blankenfeldt et al., 2006) (PDB: 2FXM) was manually placed at the interface between the two regulatory light chains for its speculated position and orientation. Surface models were generated with the “molmap” command in Chimera at a resolution of 15 Å.

#### Analysis of the preferred binding positions of myosin heads on a thin filament

In order to investigate the relation between myosin binding and the orientation of their bound actin subunit relative to the neighboring thick filament, the corresponding angle of each actin position relative to the first actin subunit in the thin filament was calculated based on actin helical parameters determined from the averaged structure. The angles where there was a myosin bound were divided into three groups depending the original thick filaments and plotted as a circular histogram. Values of angles from different filaments were combined by aligning the average of one myosin group (represented as red color in Figure 4), resulting a final histogram representing all myosin-bound actin positions (Figure 4).

In addition, the molecular model of each thin filament was converted to a myosin-binding profile sequence consisting of R, G, B, and E representing actin bound by myosin from three different thick filaments and non-bound actin, respectively. The sequences of all 30 annotated filaments were aligned by multiple sequence alignment using the “msa” package in R with the MUSCLE algorithm (Edgar, 2004) utilizing a customized weighting matrix (Figure S5; Table S3). When a thin filament was treated as a single strand, adjacent actin subunits had a huge change in orientation (166.6°) and thus very different preference for myosin binding, resulting in nonoptimal multiple sequence alignment. Therefore, each thin filament was considered as two actin strands and only one strand was used for multiple sequence alignment. The other strands were aligned using the same alignment as the first strands. The occurrence of myosin binding at each actin position was summed up for myosin from each thick filament and then used to color actin filament models in Chimera, showing averaged hotspots for myosin binding on a thin filament (Figure S5B).

To further investigate whether myosin prefers to bind at shorter distances between thin and thick filaments, the distances between thin and thick filaments at the positions where there is a myosin head bound and where myosin is absent were calculated using a python script. For each actin subunit not bound by a myosin head, three measurements were taken between the actin and three different neighboring thick filaments. For an actin subunit that is bound by a myosin head, the distance between the actin subunit and the corresponding thick filament is considered as the myosin-bound measurement while the distances between this actin subunit and the other two neighboring thick filaments are considered as the myosin-free measurements. All measurements were plotted in a histogram (Figure 4C).

#### Sub-volume averaging of the I-band thin filaments

Thin filaments in the I-band were picked automatically as described in the previous sections with crYOLO. 15,153 sub-volumes from 4 tomograms from multiple myofibrils showing I-band in the field of view were aligned with calculated priors (phi and theta angles) and refined in 3D using RELION. Helical processing was used to minimize missing wedge artifacts using the helical parameters determined in the A-band thin filament structure. This resulted to a final structure of I-band thin filament without troponin with an estimated resolution of 10.6 Å using the 0.143 criterion. The map was sharpened with a B-factor of −600 and filtered to the nominal resolution. Thin filament segments decorated by troponin densities were picked manually in IMOD. In total, 704 sub-volumes were aligned to an initial reference generated by averaging all sub-volumes without alignment in PEET. The averaged structure of the I-band thin filament including troponin has an estimated resolution of 19.8 Å after masking. A homology atomic model of the I-band thin filament was calculated using SWISS-MODEL based on the atomic model of isolated cardiac thin filament (Yamada et al., 2020) and rigid-body fitted into the averaged maps (Figures 6 and S6).

To investigate the location where the transition of tropomyosin from the M-state to C-state occurs, three tomograms containing both A-band and I-band were analyzed. Particles around the A/I junction in these tomograms were divided into 37-nm sections. Four sections (one in A-band and three in I-band) were selected using a customized script. Sections 1-4 contains 1206, 1126, 988 and 947 particles, respectively. The particles from the four sections were averaged separately using the same strategy as described above (Figures S6D and S6F). The averages were aligned to an undecorated actin filament structure for the comparison of tropomyosin positions. The particles colored by their tropomyosin states were visualized in the 3D view in IMOD to depict their distribution (Figure S6E).

#### Analysis of I-band and Z-disc organization

Different Z-disc thicknesses were observed in different sarcomeres from multiple myofibrils. The thickness was measured between the sites where α-actinins start to bind to the antiparallel thin filaments using IMOD (Figures 7A, 7D, and S7A). The relative angle between α-actinin and actin was measured as the obtuse angle between the pointed end of actin filament and α-actinin using Fiji (Figure S7A). From our observations, the Z-discs from the same myofibril have a similar thickness.

A tomogram showing both I-band and Z-disc was 4x binned and filtered initially with a SIRT-like filter using IMOD, followed by a low-pass filter at 60 Å. Thin filaments in the tomogram were first picked automatically as described in the previous section. The segments in the Z-disc were further curated manually and merged into the originally picked filaments. The polarities of the filaments were determined based on the location of the ends of the filaments. 3D organization of these filaments was generated in Fiji and shown in Chimera (Figure 6F). To analyze the organization of α-actinin in the thin-form Z-disc, potential positions of α-actinin were sampled between ends of filaments with opposite polarities. The exact location of α-actinin was searched for by aligning sub-volumes extracted at these positions to a cylinder using a limited search range with PEET. The new positions and the corresponding orientation of sub-volumes representing the location of α-actinin were clustered based on the determined new position. Obvious false-positive and false-negative positions were then curated manually. In the thick-form and intermediate Z-discs, 12 α-actinins were picked in each tomogram manually in IMOD. The angles between the pointed end of actin and the long axis α-actinin and the lengths of α-actinins in both types of Z-discs were calculated and plotted as histograms using python scripts. The 3D organization of the thin-form Z-disc was generated in Fiji and shown in Chimera (Figures 7H and 7I). A local 3D model of one thin filament with its neighboring thin filaments and α-actinins was generated by fitting models of actin filament and plotting back a map from the crystal structure of α-actinin (Ribeiro et al., 2014) (PDB: 4D1E) according to their 3-dimensional positions and orientations using tools developed as part of the TEMPy software package (Farabella et al., 2015) (Figure 7K).

Sub-volumes at the position of α-actinin were extracted from the 4x binned tomogram with a box size of 80 pixels (560 Å). An initial reference was obtained by averaging all 384 sub-volumes without refinement using PEET based on their orientation calculated from the coordinates of the α-actinin and the connected thin filaments. These sub-volumes were aligned to this reference during 3D refinement using RELION to generate a final average. The central rod domain from the crystal structure of α-actinin (PDB: 4D1E) was fitted into the averaged density manually. The actin binding domain, together with actin filaments was fitted into the density based on the atomic model of actin filament decorated by the first calponin homology (CH1) domain of filamin A (PDB: 6D8C), which shares a similar structure to the CH1 domain of α-actinin (Figures 5L and S7F).

### Quantification and statistical analysis

Statistical parameters are reported in figures and figure legends.
