# Supplementary material for: The molecular basis for sarcomere organization in vertebrate skeletal muscle
Source: Cell. 2021 Apr 15;184(8):2135–2150.e13. doi: 10.1016/j.cell.2021.02.047 (PMC8054911; doi:10.1016/j.cell.2021.02.047)
Supplement: Document S1. Tables S1–S3 [file mmc1.pdf]

Cell, Volume *184*

Supplemental information

The molecular basis for sarcomere organization

in vertebrate skeletal muscle

Zhexin Wang, Michael Grange, Thorsten Wagner, Ay Lin Kho, Mathias Gautel, and Stefan Raunser

	Ion beam voltage	Ion beam current	Thickness of lamella
**Step 1**	30 kV	500 pA	3 µm
**Step 2**	30 kV	300 pA	1 µm
**Step 3**	30 kV	100 pA	500 nm
**Step 4 (polishing)**	30 kV	50 pA	30-150 nm

Table S1. Milling strategy during lamellae production. Related to Figure S1 and STAR Methods

**Parameter**	Actomyosin complex (EMDB-12289)	re-centered myosin double-head (EMDB-12291)	I-band thin filament (without troponin) (EMDB-12292)	I-band thin filament (with troponin) (EMDB-12293)
**Data collection and processing**				
Magnification	81000	81000	81000	81000
Voltage (kV)	300	300	300	300
Exposure dose (e^-/Å^2)	130-155	130-155	130-155	130-155
Defocus range (µm)	2.4-3.4	2.4-3.4	3.3-4	3.3-4
Pixel size (Å)	1.76	1.76	1.76	1.76
No. of tomograms	8	8	4	4
Initial no. of particles	32,421	32,421	15,153	704
Final no. of particles	18,090	4,519	15,153	704
Symmetry imposed	C1	C1	Helical (-166.6° twist, 27.9 Å rise)	C1
FSC threshold	0.143	0.143	0.143	0.143
Map resolution (Å)	10.2	15.1	10.6	19.8

Table S2. Cryo-ET data collection and sub-tomogram averaging statistics. Related to STAR Methods

Score	R	G	B	E
**R**	10	-1	-1	-1
**G**	-1	0	-1	-1
**B**	-1	-1	0	-1
**E**	-1	-1	-1	0

Table S3. Weight matrix used for multiple sequence alignment with MUSCLE algorithm. Related to Figure S5 and STAR Methods
